# Research advances and application prospects of CAR-T therapy in the treatment of age-related diseases

**DOI:** 10.3389/fimmu.2026.1835582

**Published:** 2026-06-18

**Authors:** Xiaoge An, Tingting Wu, Rong Gou, Yudong Fang

**Affiliations:** 1 Department of Nephrology, The First Affiliated Hospital of Zhengzhou University, Zhengzhou, China; 2 Research Institute of Nephrology, Zhengzhou University, Zhengzhou, China

**Keywords:** age-related diseases, CAR-T therapy, cellular senescence, immune senescence, immunotherapy, kidney diseases

## Abstract

Cellular senescence constitutes the core biological basis of body aging. It not only directly drives the occurrence and progression of multiple age-related diseases, but also establishes and maintains a chronic inflammatory microenvironment through the senescence-associated secretory phenotype (SASP), thereby continuously exacerbating tissue functional decline. In recent years, CAR-T cell therapy, as the first revolutionary therapy in cancer immunotherapy, has opened up new ways to intervene in age-related diseases with its excellent target elimination capabilities. This article first studies the molecular mechanisms of cellular senescence and its pathological effects. Then a systematic overview of the design principles, development trajectory and current applications of CAR-T technology is given, focusing on the latest experimental and clinical advances in aging-related cancers, neurodegenerative diseases and cardiovascular diseases. We also dive into key current challenges, including immune-senescence, target reliability, and treatment safety. Finally, we will explore the future optimization direction of CAR-T therapy and its translational potential through various strategies such as engineered immune cells and combination therapy, hoping to provide valuable insights into research and clinical practice in this field.

## 1 Introduction

**Figure 1:**
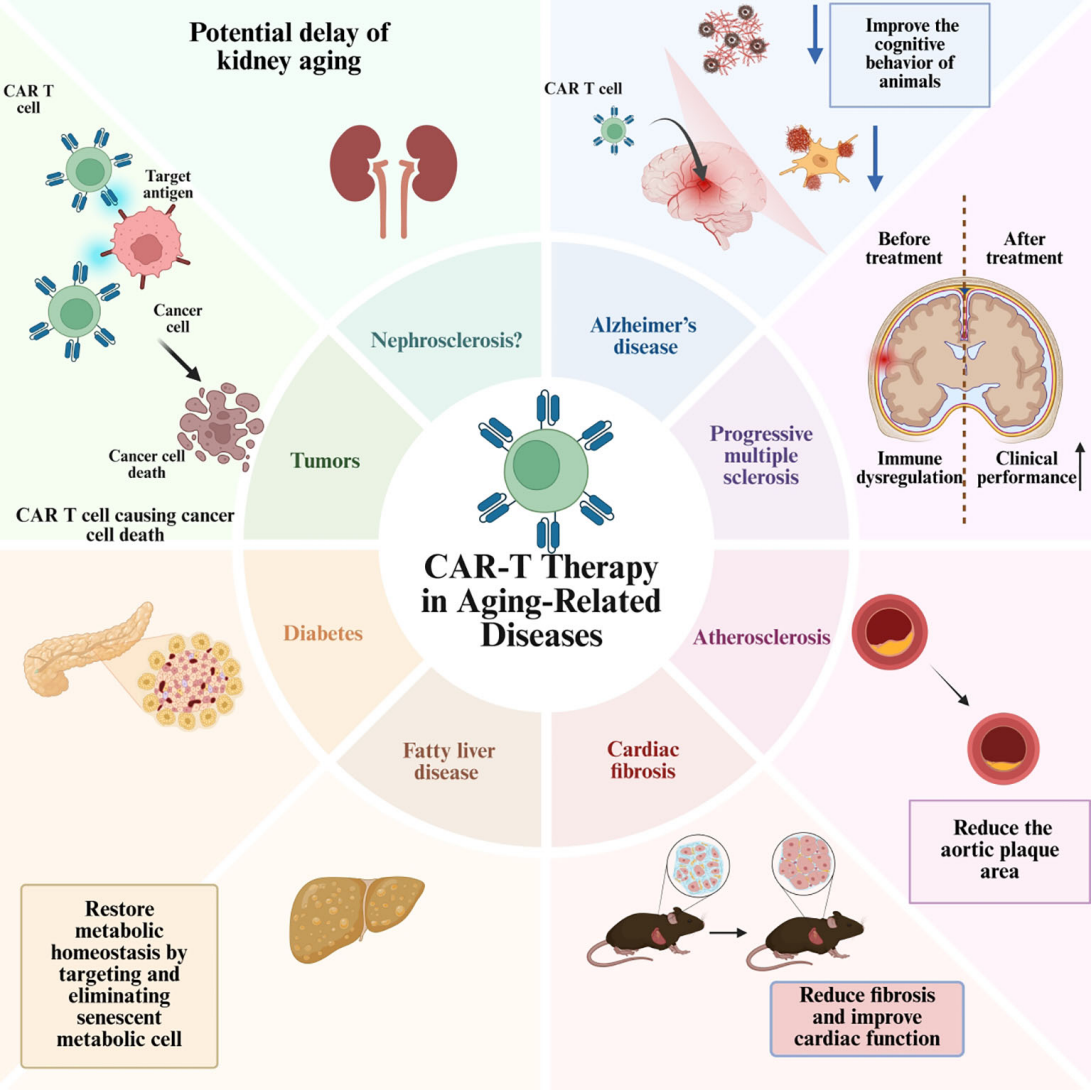
Applications of CAR-T therapy in aging-related disorders. Cancer (tumors): CAR-T cells recognize surface antigens on cancer cells and induce tumor-cell death. Kidney disease (nephrosclerosis): CAR-T therapy holds promise for slowing the progression of renal-function decline. Alzheimer’s disease: Elimination of aberrant cells improves cognitive behavior in animal models, post-treatment reductions in cerebral immune dysregulation and improved clinical status have been reported. Progressive multiple sclerosis: CAR-T therapy attenuates immune dysregulation and alleviates clinical symptoms. Heart failure / cardiac fibrosis: Decreases aortic plaque area, lowers fibrotic burden, and improves cardiac function. Fatty liver disease: Reduces hepatic fibrosis and ameliorates metabolic function. Diabetes: Targets and clears senescent metabolic cells, restoring metabolic homeostasis. 有潜在potentiol.

The global population is aging at an unprecedented rate. At the same time, the incidence of age-related diseases such as cancer, Alzheimer’s disease, and cardiovascular disease continues to increase, which poses severe challenges to the medical system and socioeconomic stability. (1, 2). Cellular senescence is a key factor in aging and age-related diseases (3). The core features of cellular senescence are irreversible cell cycle arrest and release of the senescence-associated secretory phenotype (SASP) (4–6).This process not only directly damages the self-renewal and repair capabilities of tissues, but also establishes a persistent inflammatory microenvironment that accelerates the aging of surrounding normal cells, thereby leading to the occurrence and progression of various diseases (7). Eliminating or modulating senescent cells could extend healthy lifespan and reduce the incidence of age-related diseases in laboratory animals, study shows (8). For a long time, the mainstream anti-aging strategies have mainly been to regulate metabolic pathways or use senescent cell clearance drugs to clear senescent cells. However, these methods often lack specificity and are associated with significant side effects, thus limiting their clinical application. In recent years, the emergence of CAR-T cell therapy has provided a new approach to address this challenge (9, 10). CAR-T therapy genetically engineers T cells to express a chimeric antigen receptor (CAR). This receptor can specifically recognize tumor antigens and has shown significant efficacy in a variety of hematological malignancies (such as acute lymphoblastic leukemia and diffuse large B-cell lymphoma). (11). Its core advantage lies in its precise identification and removal of target cells. This property has inspired researchers to explore its potential applications in age-related diseases. This article systematically reviews the latest research progress of CAR-T therapy in the treatment of age-related diseases. We started from the molecular mechanism of aging and integrated the technical principles and development history of CAR-T therapy. This article also provides an outlook on the clinical transformation potential and challenges of CAR-T (**Figure 1**).

## 2 Molecular mechanisms and pathological effects of cellular senescence

When cells encounter various stressors such as DNA damage, progressive telomere shortening, or oxidative stress, they often enter an irreversible state of growth arrest, commonly referred to as cellular senescence (12, 13). At this stage, cells not only stop dividing but also undergo morphological changes, a gradual functional decline, and massive release of the senescence-associated secretory phenotype (SASP) (14). The continuous accumulation of these senescent cells is considered a key driver of systemic aging and related diseases. The entire regulatory network is highly complex, involving cross-interactions among multiple signaling pathways. Its pathological effects arise both from the intrinsic dysfunction of senescent cells and from their paracrine disruption of the surrounding tissue microenvironment, ultimately compromising healthy tissue homeostasis (15).

### 2.1 Molecular regulatory mechanisms of cellular senescence

#### 2.1.1 Telomere attrition and telomerase regulation

Telomeres are located at the ends of chromosomes and play a crucial protective role. Telomere length is regarded as a biological clock, reflecting cellular age and health status. As cells undergo repeated divisions, telomeres gradually shorten. Once they reach a critical threshold, the DNA damage response (DDR) pathway is activated, prompting cells to enter a senescent state (16). Furthermore, numerous epidemiological studies indicate that telomere attrition is significantly correlated with aging, morbidity, and mortality (17). Telomerase can extend telomeres through its reverse transcriptase activity. However, in most somatic cells, this enzyme remains repressed, it is highly expressed only in stem cells, germ cells, and certain tumor cells (18).

In HIV-infected individuals, markedly shortened telomeres are observed in peripheral blood T cells. This change positively correlates with increased CD57+CD8+ T cell proportion, a hallmark of immune senescence, suggesting telomere attrition likely represents a key mechanism underpinning accelerated aging in HIV infection (19, 20). In Alzheimer’s disease (AD) mouse models, reduced telomerase activity within neurons leads to further telomere shortening, accelerating neuronal senescence accompanied by cognitive decline. Conversely, telomerase overexpression delays this pathological process (21).

#### 2.1.2 Core roles of p53-p21 and p16-Rb pathways

The p53-p21 and p16-Rb pathways represent two of the most critical regulatory pathways in cellular senescence, working in concert to maintain stable cell cycle progression. When cells encounter stress signals such as DNA damage or oxidative stress, the ATM/ATR kinase is activated, promoting the phosphorylation and nuclear translocation of p53 (tumor protein P53). This subsequently drives the expression of its downstream target gene p21 (a CDK inhibitor (22).p21 effectively blocks progression into the S phase by inhibiting cyclin-CDK complex activity, thereby inducing senescence. Meanwhile, p16 suppresses CDK4/6 activity, preventing phosphorylation of the Rb protein and inhibiting E2F transcription factor function, ultimately leading to cell cycle arrest (6).

Abnormal activation of these two pathways is prevalent in various age-related diseases. For instance, peripheral blood mononuclear cells from HIV-infected individuals exhibit markedly elevated p53 and p21 expression levels, closely associated with increased cardiovascular disease risk (23). In animal models of liver fibrosis, hepatic stellate cell senescence depends on activation of the p16-Rb pathway, while inhibiting p16 expression significantly reduces the accumulation of senescent cells, thereby alleviating the fibrotic process (24). Δ133p53 isoforms serve as effective regulators of the p53 pathway, governing critical functions in cancer, physiological and premature aging, neurodegenerative diseases, immunity and inflammation, and tissue repair. Recent research revealed that Δ133p53α counteracts the pro-aging effects induced by full-length p53 proteins, helping cells maintain proliferative capacity (25). 

#### 2.1.3 Regulation and effects of the senescence-associated secretory phenotype

**Figure 2:**
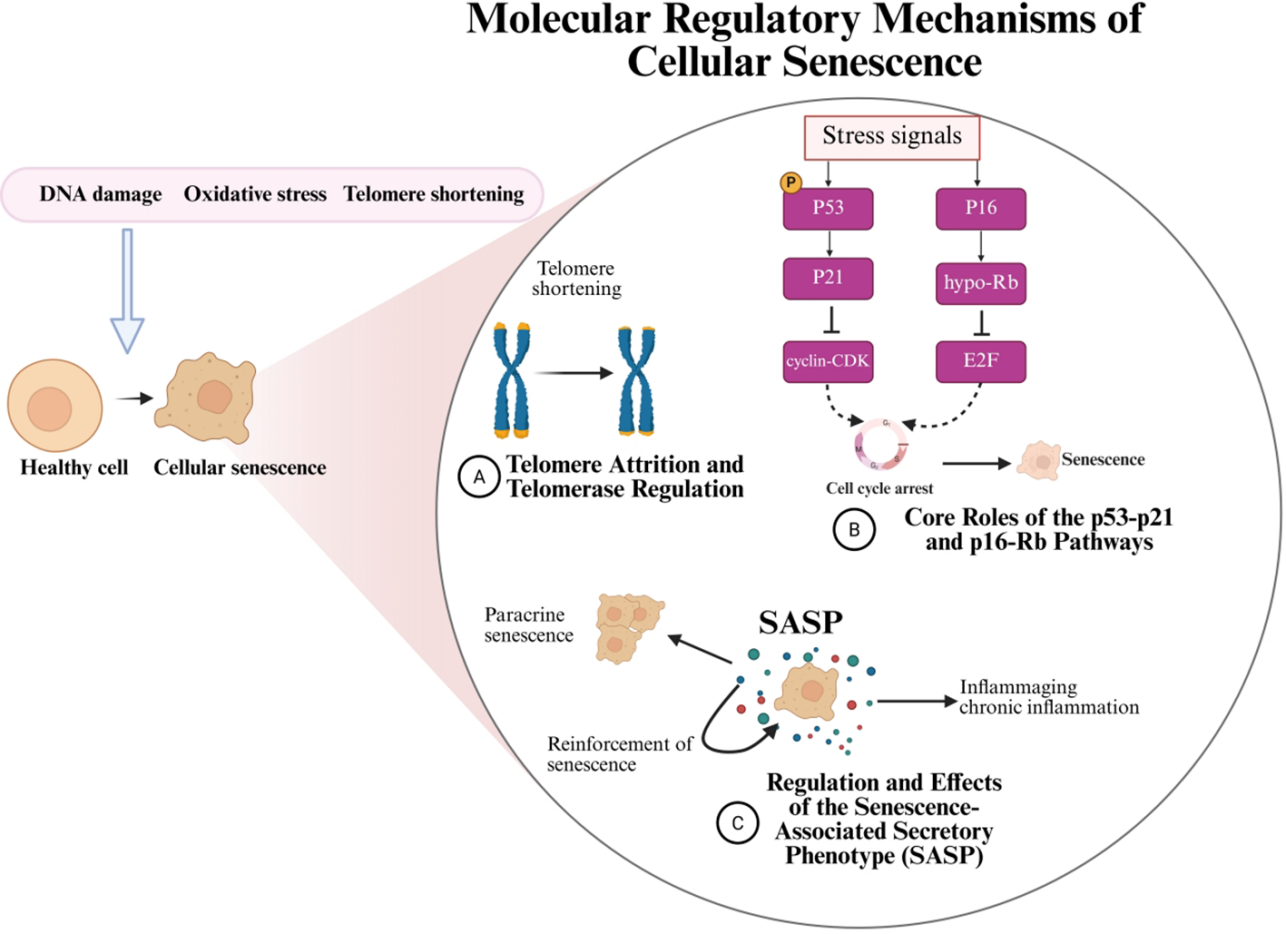
Molecular circuitry driving cellular senescence. Healthy cells exposed to DNA damage, oxidative stress, telomere erosion or other insults transit into a senescent state. **(A)** Telomere attrition and telomerase control Telomeres (chromosome-end structures) progressively shorten, propelling cells toward senescence. **(B)** Central role of the p53-p21 and p16-Rb axes Stress signals activate p53 → p21 and p16 → hypophosphorylated Rb (hypo-Rb) → E2F pathways; both routes inhibit cyclin-CDK complexes, impose cell-cycle arrest and trigger senescence. **(C)** Regulation and impact of the senescence-associated secretory phenotype (SASP) Senescent cells release SASP factors that reinforce their own senescence (autocrine loop) and, via paracrine action, propagate senescence to neighboring cells; SASP additionally fuels chronic low-grade inflammation (inflammaging).

One of the most striking features of senescent cells is their release of a complex array of factors. These factors are collectively known as the senescence-associated secretory phenotype (SASP). SASP is not a single molecule, but a complex mixture, including inflammatory cytokines such as IL-6 and IL-8 (26), chemokines such as CXCL9 and CCL2, and various matrix metalloproteinases (MMPs). They influence the microenvironment surrounding cells through paracrine effects. For example, members of the TGF-β family, vascular endothelial growth factor (VEGF), and chemokines such as CCL2 and CCL20 can propagate senescence to adjacent normal cells. This process is called paracrine senescence (27). In addition, SASP factors (such as IL-6) can act on senescent cells themselves through an autocrine mechanism, promoting cell cycle arrest and strengthening their senescent state (28). The SASP comprises numerous pro-inflammatory factors (e.g., IL-1, IL-6, IL-8, MCP-1), which can persistently activate the immune system, leading to localized or systemic chronic inflammation. This phenomenon is called “inflammatory aging”. This chronic inflammation not only promotes the accumulation of senescent cells, but is also closely associated with various age-related diseases (e.g., atherosclerosis, diabetes, and neurodegenerative diseases) (29). The expression of SASP is finely regulated by multiple signaling pathways, among which the NF-κB and MAPK pathways are particularly important (30, 31). Specifically, sustained activation of NF-κB is thought to be a core driver of initiation and maintenance of SASP (32). The effect of SASP is affected by time: in the short term, it promotes the recruitment of immune cells to clear senescent cells and exert beneficial physiological functions (33, 34). However, in the long term, the chronic inflammatory state it induces will “contaminate” the surrounding environment, prompting normal cells to enter a senescent state and driving pathological changes such as tissue fibrosis. (35). Recent research also points to metabolic regulation. A study using metabolomics showed that efficient SASP secretion relies on mitochondrial metabolic reprogramming. Inhibiting the activity of pyruvate dehydrogenase kinase (PDK) in mitochondria significantly attenuates the release of SASP, which provides a new potential therapeutic target for SASP-related diseases (36) **Figure 2**.

### 2.2 Pathological effects of cellular senescence and age-related diseases

#### 2.2.1 Bidirectional regulation between aging and tumors

Aging has a dual effect on tumors, both inhibiting and promoting their progression. Although it can prevent the uncontrolled proliferation of potential cancer cells. However, over time, the accumulation of senescent cells and the release of SASP may reshape the tumor microenvironment (TME), induce angiogenesis and lead to immune suppression. Ultimately, this promotes tumor evolution and metastasis (37) (38). When cells encounter carcinogenic stimuli (such as DNA damage, oncogene activation), the senescence program can be initiated. Through pathways such as p53-p21 and p16-Rb, these programs induce stable cell cycle arrest, blocking the unlimited proliferation of potentially cancerous cells and forming the first “natural barrier”. At the same time, certain SASP factors (such as IL-15, CXCL1) can recruit and activate macrophages, NK cells, and T cells to enhance immune surveillance to help eliminate precancerous cells (38). However, in chronic lymphocytic leukemia (CLL), the expression of senescence-related markers (such as CD28 and CD57^+^) on the surface of patients’ T cells is significantly elevated. The immune function of these cells is impaired, so the ability to monitor and eliminate cancer cells is weakened, thereby promoting tumor escape (39, 40). In solid tumors such as prostate cancer and glioblastoma, senescent fibroblasts in the tumor microenvironment continue to secrete SASP factors, including IL-6 and MMP9. These molecules powerfully drive tumor cell invasion and distant metastasis (41).

#### 2.2.2 Aging and neurodegenerative diseases

It is well established that progressive neuronal loss and pathological protein aggregation represent two core pathological alterations in neurodegenerative diseases such as Alzheimer’s disease (AD) and Parkinson’s disease (PD) (42). Increasing evidence indicates that senescence occurring in multiple brain cell types, including neurons and microglia, likely serves as a key driver of disease progression. For instance, in AD mouse models, β-amyloid (Aβ) not only directly induces neuronal senescence but also triggers senescence responses in microglia. These senescent microglia, on one hand, secrete large amounts of inflammatory SASP factors such as TNF-α and IL-1β, exacerbating the neuroinflammatory environment. On the other hand, their ability to clear Aβ is significantly diminished. Such dual effects creates a vicious cycle, senescence-inflammation-protein deposition, that continuously drives disease progression (43). This cycle similarly applies to PD. Recent research indicates that senescence is the primary risk factor for PD, with mechanisms driving senescence promoting neurodegeneration in the disease. In PD animal models, aged microglia exhibit hyperactivated NF-κB signaling, releasing SASP factors like IL-1β and TNF-α that in turn exacerbate DA neuron apoptosis (44). Future interventions targeting this cycle in the preclinical stage, such as senescent cell clearance, restoration of protein homeostasis, or energy metabolism reprogramming, hold promise for simultaneously delaying the onset and progression of both AD and PD. The MS (multiple sclerosis) phenotype is closely linked to chronological age and immune senescence. Physiological aging and inflammation-induced cellular senescence lead to oligodendrocyte pathology in inflammatory demyelinating diseases like MS, suggesting that age drives disease progression (45). Recent studies also indicate that in HIV-associated neurocognitive disorder (HAND), the HIV-1 Tat protein binds to TLR7 receptors within microglial endosomal lysosomes, triggering lysosomal damage and ultimately inducing cellular senescence (46).

#### 2.2.3 Senescence and cardiovascular disease

Cardiovascular disease has become the main cause of death in the elderly population, and more and more studies have shown that the aging of vascular endothelial cells and smooth muscle cells plays a key role in this process (47). HIV-infected patients receiving long-term combination antiretroviral therapy have significantly increased risk of cardiovascular disease (48). This additional risk may be due to certain nucleoside reverse transcriptase inhibitors (NRTIs). These drugs interfere with endothelial cell mitochondrial function, accelerate telomere shortening, and ultimately promote cellular senescence (49). For example, drugs such as tenofovir can inhibit mitochondrial DNA polymerase gamma activity, leading to accumulation of mitochondrial DNA mutations and increased levels of oxidative stress. These changes activate the p53-p21 signaling pathway and induce cellular senescence (6, 50). Notably, supplementation with mitochondria-targeted antioxidants (such as MitoQ) can partially alleviate drug-induced endothelial dysfunction, thereby delaying cellular senescence, suggesting its potential therapeutic value. (51). Senescent macrophages within plaques also play a deleterious role during atherosclerosis progression. By secreting SASP factors such as IL-6 and CXCL10, they continuously recruit more inflammatory cells and promote lipid deposition, thus exacerbating plaque instability (52). Although aging itself does not cause heart failure, age-related changes likely lower the threshold for manifesting heart failure signs and symptoms. Accumulation of DNA damage and telomere attrition results in an increase in cellular senescence and apoptosis, resulting in a decrease in the number and function of cells, contributing to the overall tissue and organ dysfunction (53).

#### 2.2.4 Aging and kidney disease

As we age, the number of senescent cells in the kidneys increases, and age-related kidney diseases become increasingly common. In patients with end-stage renal disease, signs of aging kidney function appear earlier and are more pronounced than in healthy individuals (54). It now appears that cellular senescence in kidney cells is likely to be the core mechanism driving the aging of the entire organ. Cellular senescence may not only play a role in kidney aging but may also be involved in the pathogenesis of kidney disease (55). However, it is important to note that aging itself does not directly cause kidney disease; rather, the structural and functional changes that occur in the kidney during aging may increase susceptibility to kidney disease. Existing data suggest that cellular senescence is both a marker and driver of chronic kidney disease. (56). For example, renal tubular epithelial cell senescence is a key cellular event in the progression of acute kidney injury (AKI). It promotes the transformation of AKI to chronic kidney disease through SASP. Recent studies have further shown that tubular epithelial cell senescence accelerates the progression of renal fibrosis (57). Research also suggests that cellular senescence plays an important role in metabolism-related kidney disease. Although the exact causes and mechanisms of diabetic nephropathy remain unclear. However, existing evidence has shown that podocyte senescence plays a key role and is expected to become a new target for early intervention in chronic kidney disease (58). Preclinical studies have shown that senolytics that selectively eliminate senescent cells reduce renal fibrosis and maintain renal function in experimental models of fibrosis in the kidney and other organs. This suggests that cellular senescence may become a novel therapeutic target for kidney disease (54). Therefore, early detection and intervention are crucial to control the impact of cellular senescence on kidney disease. Future research should focus on developing more biomarkers that can detect signs of cellular aging in kidney tissue, with a view to enabling early intervention and treatment.

## 3 Technical principles and development history of CAR-T therapy

Chimeric antigen receptor T-cell (CAR-T) therapy represents a breakthrough in immunotherapy. This technology employs genetic engineering to modify a patient’s own or donor-derived T cells, causing them to express an artificially designed receptor, the CAR molecule (59). This receptor typically comprises a single-chain variable fragment (scFv) that recognizes specific antigens, a transmembrane domain, and an intracellular signaling domain responsible for activating T cells, thereby enables precise identification and elimination of cells bearing the target antigen (11). Since the world’s first CAR-T therapy drug, Kymriah, was approved in 2017 for pediatric acute lymphoblastic leukemia (60), this technology has demonstrated remarkable efficacy across multiple hematologic malignancies, including lymphoma and multiple myeloma. Its application scope continues to expand, from initial use in blood cancers to solid tumors, autoimmune diseases, and even showing potential therapeutic value in aging-related diseases in recent years.

### 3.1 Technical principles and structural optimization of CAR-T therapy

#### 3.1.1 Structural composition of CAR molecules

The structure of a CAR molecule usually contains three functional components: an extracellular antigen-binding domain, a transmembrane domain, and an intracellular signaling domain (61, 62). First-generation CARs contained only the basic CD3ζ signaling domain, capable of initiating T cell receptor signaling but with limited activation efficacy and insufficient cytotoxicity against senescent cells (63). Second-generation CARs incorporated costimulatory molecules (CSMs) such as CD28 or 4-1BB, establishing a dual signaling pathway mediated by CD3ζ and CSMs, significantly enhanced T-cell proliferation and cytotoxic effects (64, 65). Currently the gold standard platform in senescence research. Both CD28 and 4-1BB co-stimulatory domains have been validated in preclinical models. For example, uPAR-targeted second-generation CAR-T cells (28ζ) demonstrated robust senolytic activity in liver fibrosis and age-related metabolic dysfunction models (66) (67). NKG2D-CAR-T cells (BBζ) effectively eliminated senescent neurons and glial cells in neurodegenerative disease models (68).Third-generation CARs further fuse two costimulatory molecules (e.g., CD28 and 4-1BB in series), thereby extending CAR-T cell survival in vivo. (69, 70).It shows enhanced T-cell expansion and persistence but may accelerate T-cell exhaustion in the chronic inflammatory environments typical of aging (71). Fourth-generation CARs, also known as TRUCKs, incorporate a cytokine-inducing domain that promotes cytokine production after antigen recognition. (70, 72, 73).It can have dual functions of target cell killing and cytokine secretion, but carry significant inflammatory risks in elderly populations (71). Fifth-generation CARs build upon the second-generation design by incorporating a truncated cytoplasmic IL-2 receptor β chain (IL-2Rβ) domain, activating the JAK-STAT signaling pathway. (74, 75).It can incorporate IL-2Rβ signaling to enhance T-cell stemness and metabolic fitness, which is particularly advantageous for overcoming immune senescence in elderly patients (76).

Different costimulatory signals significantly affect the aging behavior of CAR-T cells. CAR-T cells expressing the 4-1BB costimulatory domain (BBζ) exhibit increased p16 expression and impaired proliferation after repeated antigen stimulation, which are hallmarks of aging. In contrast, CAR-T cells using the CD28 costimulatory domain (28ζ) showed better anti-aging stability. (77).

#### 3.1.2 CAR-T cell preparation workflow and optimization

The standard CAR-T cell preparation process usually begins with collecting peripheral blood from patients or healthy donors, followed by obtaining peripheral blood mononuclear cells (PBMCs) through leukapheresis. Next, anti-CD3/CD28 antibodies or immunomagnetic beads are used to activate T cells in vitro. Subsequently, the CAR encoding gene is introduced into T cells through viral vectors such as lentivirus or retrovirus, or non-viral methods such as transposon systems and mRNA electroporation. Under the stimulation of cytokines such as IL-2, IL-7 and IL-15, these engineered T cells undergo large-scale expansion. Finally, after the patient receives appropriate lymphocyte depletion pretreatment, CAR-T cells are reinfused to complete the treatment (78, 79).

**Figure 3:**
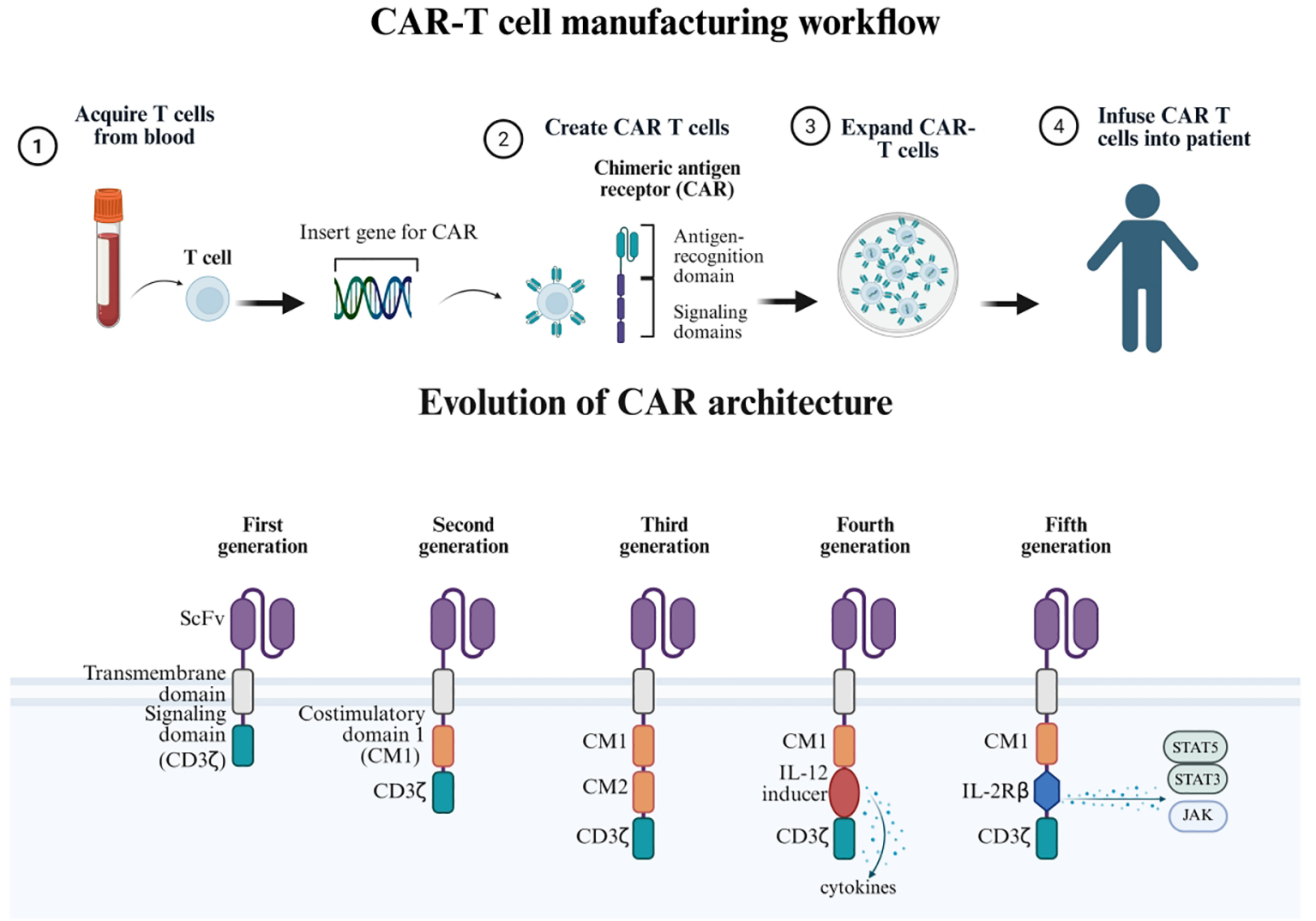
CAR-T cell manufacturing workflow. 1. T-cell acquisition: isolate autologous T cells from the patient’s peripheral blood. 2. CAR construction: introduce the chimeric-antigen-receptor (CAR) gene into T cells; the encoded CAR comprises an antigen-recognition domain plus signaling domains. 3. Expansion: culture the genetically modified T cells ex vivo to achieve clinically relevant numbers. 4. Infusion: reinfuse the expanded CAR-T product into the patient. Evolution of CAR architecture. All CAR generations share an extracellular scFv (antigen-recognition), a trans-membrane spacer, and intracellular signaling modules; they differ in the number and type of co-stimulatory/signaling domains: 1st generation: CD3ζ only. 2nd generation: CD3ζ + one co-stimulatory domain (e.g., CM1). 3rd generation: CD3ζ + two co-stimulatory domains (e.g., CM1 + CM2). 4th generation (TRUCK): 3rd-gen backbone + an inducible cytokine cassette (e.g., IL-12) that secretes immune-enhancing cytokines. 5th generation: 3rd-gen backbone + a cytokine-receptor signaling domain (e.g., IL-2Rβ) enabling JAK/STAT pathway activation.

In recent years, researchers have continuously optimized preparation strategies to improve the efficacy and durability of CAR-T products, with special emphasis on delaying cell aging. For example, using IL-21 to replace traditional IL-2 in in vitro culture helps CAR-T cells maintain a metabolic state dominated by oxidative phosphorylation (OXPHOS) and reduce their dependence on glycolysis. This approach reduces the expression of aging markers such as CD57 and p21, thereby delaying functional decline (80). In addition, CAR-T cells prepared using mRNA electroporation technology can achieve transient CAR expression. Although this method has a short duration of action, it can effectively avoid cell exhaustion and aging caused by long-term antigen stimulation (81). This type of “short-term expression” CAR-T cells has been proven to be safe and effective in clearing senescent cells in animal studies, providing a new strategy for clinical application. (82). Recent research indicates that aquaporin-mediated transfection yields 1.7 to 2 times higher CAR-T cell production compared to electroporation, with superior cell viability and recovery rates (83). Zhang et al. also developed a cardiolipin mimetic phosphoramide (CAMP) lipid that can transfect T cells without antibody modification. The lipid encapsulates the CAR-encoding circular RNA, further extending the duration of mRNA expression in mouse spleens and T cells (84). **Figure 3**.

#### 3.1.3 Applicability of different CAR generations in senescent cell clearance

The design requirements for CAR-T cells targeting senescent cells differ fundamentally from those targeting tumor cells, driven by core biological differences between the two cell types. These differences directly determine the relative performance of different CAR generations in age-related disease applications.

Tumor therapy aims for long-term immune surveillance to prevent recurrence, whereas senolytic therapy requires transient, potent, and controlled cytotoxicity to avoid exacerbating chronic inflammaging in elderly populations (67) (71). Within this framework, second-generation CAR-T cells remain the current gold standard for senescence research and translation. The CD28ζ variant is the first choice for most age-related diseases, including liver fibrosis and metabolic dysfunction, owing to its rapid expansion, acute cytotoxicity, and low risk of chronic inflammation (66, 67).

To further quantify the relative advantages and disadvantages of different CAR generations in age-related diseases, we conducted a comprehensive risk-benefit analysis specifically tailored to elderly patients (**Table 1**). The data presented in this table are primarily derived from animal models and early clinical investigations, and large-scale human clinical trial data remain limited (71).

**Table 1:** Risk-benefit analysis of different CAR generations in age-related diseases.

CAR generation	Core structural features	Performance in tumor therapy	Performance in senescent cell clearance	Key risks in aging populations	Recommended application scenarios in aging diseases
1st Generation	CD3ζ intracellular domain only	Obsolete (insufficient T-cell activation)	No reported applications	Low toxicity but no therapeutic efficacy	None
2nd Generation (CD28ζ)	CD3ζ + CD28 co-stimulatory domain	Excellent for hematological malignancies; rapid response but shorter persistence	Optimal; rapid cytotoxicity, minimal off-target effects, low risk of chronic inflammation	Mild CRS (grade 1–2 in <10% of patients)	Liver fibrosis, metabolic dysfunction, cardiovascular fibrosis
2nd Generation (BBζ)	CD3ζ + 4-1BB co-stimulatory domain	Superior persistence; better for preventing tumor recurrence	Good but suboptimal; increased T-cell senescence and long-term off-target risk	Higher risk of T-cell exhaustion and delayed toxicity	Neurodegenerative diseases (requires longer persistence)
3rd Generation	CD3ζ + 2 co-stimulatory domains (e.g., CD28 + 4-1BB)	Enhanced expansion and persistence; better for solid tumors	Moderate; prolonged activation may exacerbate inflammaging	Increased risk of chronic inflammation and ICANS	Severe organ fibrosis (limited to short-term treatment)
4th Generation (TRUCKs, IL-12)	2nd generation + cytokine secretion domain (IL-12)	Effective for solid tumors; modulates immunosuppressive TME	Poor; high inflammatory risk outweighs benefits	High risk of severe CRS/ICANS; potentially fatal in elderly	Not recommended for systemic use; only for local administration with inducible expression
5th Generation	2nd generation + IL-2Rβ domain	Promising for both hematological and solid tumors	Excellent potential; overcomes immune senescence	Limited clinical data; theoretical risk of uncontrolled T-cell proliferation	Elderly patients with severe immune senescence

### 3.2 Development history and current clinical applications of CAR-T therapy

#### 3.2.1 Breakthrough advances in hematologic malignancies

The clinical application of CAR-T therapy in hematologic malignancies has reached relative maturity. Since its first clinical use in 2010, CAR-T cell therapy has demonstrated significant success in treating B-cell malignancies (85). By 2025, six CAR-T cell therapies had received approval from the FDA (U.S. Food and Drug Administration) and EMA (European Medicines Agency) globally for hematologic malignancies (11, 86). Idecalumab cell therapy (ide-cel) received FDA approval for treating relapsed refractory multiple myeloma (RRMM), becoming the first CAR-T product approved for multiple myeloma (87). Target molecules for hematologic malignancies primarily include CD19 (e.g., Kymriah, Yescarta) (88), BCMA (e.g., Abecma, Carvykti), and CD22 (89).Indications include acute lymphoblastic leukemia (ALL), diffuse large B-cell lymphoma (DLBCL), and multiple myeloma (MM) (11). Clinically, CD19-targeted CAR-T cells demonstrate high initial response rates in treating relapsed/refractory B-cell non-Hodgkin lymphoma patients, with remission rates reaching 70% to 90% (90). ssCART-19 is a novel autologous CD19-specific CAR-T therapy that incorporates shRNA technology to silence IL-6. It demonstrated favorable safety in a Phase I clinical trial for relapsed/refractory B-cell acute lymphoblastic leukemia (91). Actalycabtagene autoleucel, a novel humanized anti-CD19 CAR-T cell therapy, received Indian approval on October 13, 2023, for treating relapsed/refractory B-cell lymphoma and relapsed/refractory B-cell acute lymphoblastic leukemia, becoming the first domestically developed CAR-T product approved for marketing in India globally. refractory B-cell acute lymphoblastic leukemia (ALCL), becoming the world’s first domestically developed CAR-T product approved for marketing in India (92). Additionally, we found that the FDA-approved isocitrate dehydrogenase 2 (IDH2) inhibitor enasidenib enhances memory CAR T-cell formation and maintains anti-leukemic cytotoxicity *in vivo* (93).

#### 3.2.2 Challenges and breakthroughs in solid tumors

Compared with hematological malignancies, the application of CAR-T cell therapy in solid tumors faces greater challenges. The main reasons are the high antigen heterogeneity in solid tumors, the tumor microenvironment (TME) inhibiting CAR-T cell function, and the difficulty of CAR-T cells effectively infiltrating solid tumors (94). These challenges are significantly amplified in elderly individuals due to age-related biological changes that synergize with tumor-intrinsic factors to further reduce CAR-T efficacy:progressive stromal fibrosis with aging exacerbates CAR-T cell infiltration defects. increased myeloid-derived suppressor cell (MDSC) infiltration in the aged tumor microenvironment (TME) amplifies immunosuppression.increased myeloid-derived suppressor cell (MDSC) infiltration in the aged tumor microenvironment (TME) amplifies immunosuppression (95, 96).This combination of poor manufacturing quality and enhanced in vivo dysfunction makes solid tumor CAR-T therapy particularly challenging in the elderly population. (97, 98).

In order to break through these technical bottlenecks, scientific researchers are promoting technology optimization through various channels. Solutions for tumor antigen heterogeneity and antigen loss include bispecific CAR-T cells (99), hybrid CAR-T cells (100), CAR-T cell switching technology (101), and radiomics (AI) (102). In addition, Baker and Roybal’s team recently developed an advanced engineered receptor for soluble cell communication and disease sensing. They successfully used this technology to guide CAR-T cells to specifically target and destroy solid tumors expressing soluble disease-related factors, thereby minimizing off-target toxicity. (103). To overcome the immunosuppressive tumor microenvironment, researchers have designed CAR-T cells that secrete immunostimulatory cytokines such as IL-12, IL-18, and IL-15 (104); CAR-T cells that target regulatory T cells (Tregs), myeloid-derived suppressor cells (MDSCs) (105), and M2 macrophages; and programs that combine CAR-T cells with chemotherapy (106).We can also develop armored CAR-T cells that secrete immunostimulatory cytokines such as IL-12, IL-18 or IL-15 (such as IL-15/IL-21 autocrine circuit or CXCR2 chemokine receptor-modified CAR-T cells). These therapies maintain higher activity in the fibrotic microenvironment while promoting cross-infiltration of endogenous CD8^+^ T cells (107) (108). To enhance tumor infiltration of CAR-T cells, researchers have developed nanobody-based CAR-T therapies, CAR-T cells expressing chemokine receptors, locally administered CAR-T cells, CAR-T cells targeting stromal cell-associated antigens, CAR-T cells secreting matrix-degrading enzymes, molecular torpedoes, and CAR-T cell therapies in combination with immune checkpoint inhibitors (anti-CTLA-4 or anti-PD-1 monoclonal antibodies) (109). A recent study identified a synNotch receptor designed to bind to the brain-localized extracellular matrix protein BCAN. It can locally induce CAR expression targeting EphA2 and IL13Rα2, resulting in complete clearance of xenograft tumors derived from glioblastoma patients (110–112).

#### 3.2.3 Expansion into non-oncological applications

In recent years, the application of CAR-T therapy has expanded from the field of oncology to autoimmune diseases, chronic infections and age-related diseases (113). In autoimmune diseases, CD19-targeted CAR-T cells have been used to specifically eliminate pathogenic B cells, and clinical studies are currently underway for diseases such as systemic lupus erythematosus (SLE) and rheumatoid arthritis (RA) (114). CD19-CAR-T cells can profoundly eliminate CD19^+^ B cells within synovial tissue, including synovial infiltrating B cells that are difficult to eradicate with conventional monoclonal antibody therapy (115, 116) (117). In infectious diseases, A new technology based on CAR-T cell immunotherapy has demonstrated potential for clearing persistent infections (118). Liu et al. developed a broadly neutralizing antibody-derived (bNAb-derived) CAR-T cell therapy that can exert specific cytotoxic activity against HIV-1-infected cells, eliminate latently infected cells, and delay viral rebound (119). In age-related diseases, uPAR-targeted CAR-T cells have been shown to effectively eliminate uPAR-expressing senescent hepatocytes and activated hepatic stellate cells in the liver. The therapy significantly reduced the number of senescent cells, reduced fibrotic areas, and improved liver function, with no significant toxicity observed at low doses (67, 120).

#### 3.2.4 Expanded applications in renal medicine

The application of CAR-T in kidney diseases has expanded from tumor-associated renal impairment (121) to autoimmune nephropathy, demonstrating significant potential, particularly in eliminating pathogenic B cells and restoring immune tolerance. Compared with other therapies, CAR-T therapy for multiple myeloma (MM) with renal impairment achieves tumor cell clearance while improving renal function (122). CD19-CAR-T cells effectively targeted and eliminated CD19+ B cells in lupus mouse models, reducing autoantibody secretion, alleviating lupus symptoms, and prolonging mouse survival (123). In another clinical study by Mackensen et al., five patients who had previously failed multiple treatments were enrolled; These patients demonstrated significant reductions in proteinuria and anti-dsDNA levels, along with elevated complement levels, three months after anti-CD19 CAR-T cell therapy (124). These data collectively indicate that CD19-CAR-T cell transfer is feasible, well-tolerated, and highly effective in Lupus nephritis. Substantial evidence suggests extensive B-cell involvement in the pathogenesis of AAV (ANCA-associated vasculitis). Recently, Dörte Lodka et al. investigated the potential of CD19-CAR-T cell therapy for AAV using a preclinical mouse model of MPO-AAV. The CAR-T cells targeted CD19+ B cells and plasma cells, reducing ANCA (anti-neutrophil cytoplasmic antibody) production and preventing necrotizing crescentic glomerulonephritis. Renal fibrosis is a hallmark of chronic kidney disease. Zhao et al. demonstrated that CAR-T therapy targeting ECM-producing cells alleviates fibrosis in chronic kidney disease, with anti-fibrotic effects also validated in human kidney organs. Unfortunately, no CAR-T applications currently exist for kidney aging-related diseases (125).

## 4 Research progress of CAR-T therapy in ageing-related diseases

In 2020, Amor C et al. engineered uPAR-CAR-T cells (using urokinase receptor as a universal senescence marker) (67). In models of liver fibrosis and lung adenocarcinoma-induced senescence, a single injection can achieve long-term clearance of senescent cells and restore tissue function, directly proving for the first time that “CAR-T can clear senescent cells.” Even more exciting is that uPAR-CAR-T cells can also improve age-related metabolic disorders and physical dysfunction. It maintains its long-lasting and preventive effects by activating memory CD8 T cells and effector CD8 T cells. (66).

The core mechanism underlying age-related diseases lies in the progressive accumulation of senescent cells within the body, which mediate persistent chronic inflammation by releasing the SASP. CAR-T therapy, with its ability to precisely identify and eliminate specific target cells, offers a novel approach to intervening in such diseases: it can directly target and clear pathological senescent cells (126), or be used to eliminate age-related abnormal cells such as tumor cells or overactive reactive immune cells. Next, I will introduce the latest developments in CAR-T therapy in this field from the perspective of different disease types, drawing on significant recent research.

### 4.1 Research advances in CAR-T for ageing-related tumors

Tumorigenesis is closely linked to aging, with incidence rates significantly increasing with age (127). The tumor microenvironment usually contains a large number of senescent cells, such as senescent fibroblasts and immune cells, which promote tumor progression and immune suppression by secreting SASP.CAR-T therapy can not only selectively eliminate tumor cells, but also effectively reduce pathological senescent cells by identifying specific surface markers on these cells, thereby improving the overall treatment effect.

In hematological malignancies, patients often exhibit significant immune senescence, which is manifested by shortened telomeres, increased proportion of CD57-positive cells, and weakened function of T cells. These changes will reduce the efficacy of CAR-T cells (128). Genetic engineering of CAR-T cells can enhance their anti-aging properties. For example, overexpression of the anti-aging p53 isoform Δ133p53α in CD19-CAR-T cells inhibits senescence signaling pathways, enhances CAR-T cell function, and improves the remission rate of acute lymphoblastic leukemia (ALL) mouse models (129).

The efficacy of CAR-T cells against solid tumors remains limited, hindered by antigenic heterogeneity, immune evasion mechanisms, and the immunosuppressive tumor microenvironment (TME) (130).These challenges are particularly pronounced in elderly patients, where age-related biological changes further compound treatment disparities. This disparity was independently associated with three age-specific factors: higher intratumoral collagen content, increased MDSC infiltration, and more severe autologous T cell senescence (96, 131).Senescent fibroblasts in the solid tumor microenvironment promote tumor invasion and suppress immune responses by secreting SASP factors such as IL-6 and matrix metalloproteinases (MMPs) (132). CAR-T therapy targeting these senescent stromal cells, in combination with conventional tumor-targeted CAR-T, can improve the tumor microenvironment and enhance anti-tumor immune responses in elderly patients. (133).

### 4.2 Research progress of CAR-T in neurodegenerative diseases

Neurodegenerative diseases (NDDs), including Alzheimer’s disease (AD), Parkinson’s disease (PD), and multiple sclerosis (MS), are the leading causes of physical and cognitive impairment worldwide (134). Neuronal loss and persistent neuroinflammation are the common pathological basis of Alzheimer’s disease (AD) and Parkinson’s disease (PD). Among them, the aging of brain cells such as neurons and microglia is considered a key driver of this process (135, 136). In recent years, CAR-T therapy has emerged as a potential intervention strategy due to its precise targeting ability, but its application in NDDs remains in the very early preclinical stage with significant translational challenges.

In Alzheimer’s disease, Aβ plaques and hyperphosphorylated tau protein deposition are a significant pathological feature, causing neurons and microglia to enter a senescent state. The FDA and EMA recently approved three monoclonal antibodies against Aβ. However, they only slow cognitive decline by about 30% (137). Given the limitations of passive immune strategies in AD, cell therapy has emerged as an attractive alternative. Using mouse embryonic fibroblasts (MEFs) and astrocytes (AST) as aging models, Deng et al. demonstrated that NKG2DL expression is elevated under genotoxicity and oxidative stress. NKG2D-CAR T cells exhibited potent cytotoxicity against these senescent cells and had little effect on non-senescent cells (68).In addition, CAR-T cell therapy targeting tau protein is under research. The therapy may reduce neurofibrillary tangle formation and protect neuronal function by clearing hyperphosphorylated tau protein (138). All current senolytic CAR-T studies for AD are preclinical, and none have entered clinical trials. Although aging accelerates the progression of Parkinson’s disease (PD), there are currently no clinical studies or animal experiments that directly verify the application of CAR-T cell therapy in the treatment of PD. Multiple sclerosis (MS) is not a typical age-related degenerative disease, but its onset and progression are closely related to age-related immune dysregulation. Fischbach et al. demonstrated that CD19 CAR-T cells not only suppress inflammatory relapses in MS patients, but also eliminate disease-promoting B cells residing in the central nervous system (139). In a clinical trial by Qin et al., five patients with progressive multiple sclerosis (PMS) were treated with anti-B-cell maturation antigen chimeric antigen receptor T (CAR-T) cell therapy. They observed clinical remission in patients, long-term expansion of CAR-T cells, and reduced cell depletion in the cerebrospinal fluid. (140).These findings suggest that CAR-T therapy may have potential for immune-mediated neurological diseases associated with aging. (68).

### 4.3 Research advances in CAR-T therapy for cardiovascular diseases

Cardiovascular disease is the leading cause of death among the elderly, closely associated with the progressive aging of vascular endothelial cells, smooth muscle cells, and cardiomyocytes (47, 141).In recent years, CAR-T therapy has emerged as a new treatment method. It slows disease progression by precisely eliminating specific cells, targeting aging vascular cells or pro-inflammatory immune cells. In atherosclerosis, abnormal lipid deposition combined with chronic inflammatory responses drive plaque formation. Among them, senescent endothelial cells (142) and macrophages (143) play key roles. CAR-T cells can target uPAR expressed on senescent macrophages, inducing senescent cell death. This approach holds promise for alleviating chronic inflammation in atherosclerosis, stabilizing plaques, and delaying disease progression (52). In vitro and in preclinical mouse models, CAR-T cells can also target macrophages, reducing oxLDL uptake and foam cell formation (144). The progression of heart failure is frequently accompanied by myocardial cell senescence and progressive fibrosis. Senescent cardiac fibroblasts accelerate myocardial remodeling and functional deterioration through excessive secretion of collagen and multiple SASP factors (145). In a stress-overload-induced heart failure mouse model, these cells highly express fibroblast activation protein (FAP) (146). Application of FAP-CAR-T cells specifically eliminates senescent fibroblasts, significantly reducing cardiac interstitial collagen deposition and improving cardiac function (147). In the original study by Aghajanian et al. (148) (148), FAP-CAR-T treatment did not cause significant systemic toxicity, weight loss, or histopathological damage to normal organs in mice, despite detectable CAR-T cell infiltration into the bone marrow. Similarly, the mRNA-based FAP-CAR-T approach showed no evidence of off-target toxicity in preclinical models, likely due to the transient nature of CAR expression (149).While these preclinical results are highly promising, it is important to note that all studies to date have been conducted in small animal models, and further validation in large animal models and human clinical trials is required.

Another critical translational challenge is whether transient CAR-T cell persistence is sufficient for the management of chronic cardiovascular disease or whether repeated dosing would be required. Both the conventional DNA-based FAP-CAR-T and the mRNA-based transient CAR-T approaches produced durable improvements in cardiac function despite limited CAR-T persistence. This suggests that a single, targeted depletion of activated myofibroblasts may be sufficient to interrupt the self-sustaining fibrotic cascade and allow endogenous tissue repair mechanisms to restore cardiac function. Unlike cancer, where long-term CAR-T persistence is often required to prevent relapse, cardiac fibrosis is a progressive but potentially reversible process (150). Once the pathological fibroblast population is eliminated and the pro-fibrotic microenvironment is normalized, residual fibroblasts can return to a quiescent state, and ongoing ECM deposition is halted. However, for patients with advanced chronic heart disease or recurrent injury (e.g., repeated MIs), repeated dosing may be necessary to maintain therapeutic efficacy. The mRNA-based CAR-T platform is particularly well-suited for repeated administration because it avoids the risk of insertional mutagenesis, does not induce long-term CAR-T cell memory, and allows for precise dose titration and treatment discontinuation if toxicity occurs (151).

### 4.4 Research advances in CAR-T for metabolic diseases

Metabolic diseases such as diabetes and non-alcoholic fatty liver disease (NAFLD) are closely associated with the body’s aging process. The senescence of functional cells like adipocytes and hepatocytes often triggers insulin resistance and lipid metabolism disorders, thereby accelerating disease progression (152).In recent years, CAR-T therapy has been gradually used to intervene in metabolic aging due to its potential to precisely eliminate specific cells. Targeted elimination of senescent metabolic cells provides a new approach to restore metabolic homeostasis. Preclinical studies have shown that redirecting CAR-T cells to pancreatic beta cells can prevent beta cell destruction, thereby preventing the onset and progression of diabetes in mouse models (153).CAR-T cells targeting uPAR-positive cells improved metabolic dysfunction and glucose tolerance in aged mice and high-fat diet-fed mice (66). In NAFLD, hepatocyte senescence and abnormal activation of hepatic stellate cells are key mechanisms driving disease progression (120, 154). Studies indicate significantly elevated uPAR expression on senescent hepatocyte surfaces. Utilizing uPAR-CAR-T cells to specifically eliminate these cells markedly reduced collagen deposition and improved liver function, with no apparent toxicity observed at low doses (155). In a non-alcoholic steatohepatitis (NASH) model, m.uPAR-h.28z CAR-T cells demonstrated regulatory effects on cellular senescence, effectively reversing liver fibrosis (67). CAR-T cells targeting FAP can eliminate profibrotic hematopoietic stem cells and regulate immune cells, endothelial cells and liver cells, thereby reducing inflammation and restoring liver homeostasis, significantly reducing liver fibrosis (156).

## 5 Challenges and optimization strategies for CAR-T therapy in age-related diseases

CAR-T therapy has shown great potential in treating age-related diseases, but its practical application faces multiple challenges. Challenges include cellular dysfunction caused by immune-senescence, insufficient targeting specificity, safety risks (such as cytokine release syndrome and neurotoxicity), and complex manufacturing processes (157).

### 5.1 Patient immune senescence

A Fundamental Barrier to CAR-T Therapy for Age-Related Diseases is the impact of host aging itself on CAR-T therapy efficacy represents the most critical unaddressed challenge in translating senolytic CAR-T technology to elderly patients, who constitute the primary population affected by age-related diseases. This barrier manifests at three interconnected levels: inferior clinical outcomes in elderly cohorts, intrinsic defects in autologous T cells from aged donors (158), and impaired in vivo function of CAR-T cells in the aged immune microenvironment (159).

#### 5.1.1 Clinical trial outcomes of CAR-T therapy specifically in elderly patients

Elderly patients (≥65 years) account for >60% of newly diagnosed hematologic malignancies and nearly all age-related degenerative diseases (160, 161), yet they have been historically underrepresented in early CAR-T clinical trials. Recent dedicated analyses and large-scale real-world studies have confirmed that CAR-T therapy retains significant efficacy in this population, but with distinct safety and durability profiles.

In hematologic malignancies (the most clinically advanced indication), CAR-T therapy achieves comparable response rates in elderly and younger patients, albeit with slightly elevated toxicity risk. In the pivotal ZUMA-1 trial of axicabtagene ciloleucel for refractory large B-cell lymphoma, patients ≥65 years (n=51) achieved an overall response rate (ORR) and complete response (CR) rate, which were non-inferior to younger patients (162). The JULIET trial of tisagenlecleucel similarly reported an ORR in patients ≥65 years, with no significant difference in 12-month overall survival (OS) compared to younger cohorts (163). For relapsed/refractory multiple myeloma, the KarMMa trial of idecabtagene vicleucel showed that patients ≥65 years had an ORR of 73% and CR of 33%, nearly identical to younger patients, with comparable 12-month progression-free survival (PFS) (164). Baseline immune senescence markers were identified as independent predictors of both treatment efficacy and toxicity in elderly cohorts (165).

#### 5.1.2 Preclinical mechanisms of age-related CAR-T dysfunction

Preclinical studies have systematically elucidated how the aged host immune environment impairs CAR-T therapy at three key stages: manufacturing, in vivo expansion, and long-term persistence.

Autologous T cells from elderly individuals have intrinsic functional defects that significantly reduce manufacturing success rates and product quality (98). Elderly donors exhibit reduction in naive T cell numbers and a marked expansion of terminally differentiated CD57^+^CD28^+^ T cells. These cells are resistant to anti-CD3/CD28 activation and are more prone to apoptosis during in vitro expansion, leading to lower CAR-T production yields and higher rates of manufacturing failure (166).

The aged systemic immune environment significantly impairs the early expansion phase of infused CAR-T cells, which is a key determinant of therapeutic efficacy. Standard fludarabine/cyclophosphamide lymphodepletion conditioning achieves lower efficacy in aged mice compared to young mice, leaving higher residual numbers of regulatory T cells (Tregs) and myeloid-derived suppressor cells (MDSCs) that potently inhibit CAR-T proliferation (166, 167).

Aged hosts fail to support the development of long-lived CAR-T memory cells, leading to higher relapse rates and reduced durability of response. In aged mouse models, infused CAR-T cells preferentially differentiate into short-lived effector T cells (CD62L^+^CD44^+^) rather than central memory T cells (CD62L^+^CD44^+^), resulting in reduction in CAR-T cell numbers (168).Senescent stromal cells and M2 macrophages in aged tissues secrete high levels of TGF-β and IL-10, which induce CAR-T cell senescence and apoptosis.

#### 5.1.3 Optimization strategies to overcome age-related CAR-T dysfunction

Immune senescence represents age-related immune dysfunction characterized by reduced T cell diversity, diminished effector function, and increased expression of senescence markers. In elderly patients, autologous T cells often exhibit advanced senescence, leading to poor CAR-T cell preparation efficiency and limited expansion capacity (169).This issue is particularly critical for solid tumor CAR-T therapy, where aged T cells must not only survive ex vivo manufacturing but also function effectively in the highly immunosuppressive and fibrotic TME of elderly patients.

Three primary strategies exist for restoring the T cell repertoire: replacement, reprogramming, and rejuvenation of senescent cells (170). Corresponding improvement strategies include: utilizing allogeneic T cell sources, such as umbilical cord blood or T cells differentiated from induced pluripotent stem cells (iPSCs). These cells possess longer telomeres and lower expression of senescence markers, significantly enhancing CAR-T expansion capacity and persistence in elderly individuals (171, 172); Genetically enhancing anti-aging properties, such as overexpressing telomerase reverse transcriptase (TERT) to extend telomeres or knocking out aging-related genes like p16 (173); Restoring and maintaining thymic microenvironments through bioengineered thymic organoids combined with growth promoters and cytokines (e.g., IL-21) to reverse thymic atrophy effects. IL-21 has recently been identified as a thymic stimulatory factor that triggers thymopoiesis in aged mice, demonstrating significant immune restoration and rejuvenation of peripheral T cell pools (174, 175).

### 5.2 Target specificity and off-target toxicity

The specificity of target selection directly impacts CAR-T therapy safety. Current markers for identifying senescent cells (e.g., uPAR, NKG2DLs) are also expressed at low levels in normal tissues, potentially causing off-target toxicity. Current strategies to enhance specificity primarily include: designing bispecific CARs or multi-target CAR-T cells, including tandem CAR-T cells (176). Developing conditionally activated CAR systems, such as those utilizing hypoxia response elements (HREs), enables CAR activation exclusively in hypoxic microenvironments like tumors or fibrotic tissues, avoiding activation in normal tissues (177). Additionally, novel targets with higher senescence specificity can be explored. Another study identified the glycolipid antigen GD3 as a specific marker for senescent cells (178). GD3 CAR T cells target, limit and treat spontaneous tumor in aging Tsc2+/– mice (179). Sun et al. found that CD24-CAR-T cells blocked the CD24-Siglec-10 pathway, thereby enhancing the ability of macrophages to phagocytose and clear myeloma cells, and had better efficacy in the treatment of MM than BCMA-CAR-T cell therapy. (180) **Table 2**.

**Table 2:** Specific target selection for CAR-T therapy.

Target antigen	Animal model	Organ	Conclusion	Author
uPAR	Mouse liver fibrosis aging model;senescence model of lung adenocarcinoma in mice	Liver and lung	Ablate senescent cells	(67)
uPAR	Natural aging mouse model;Metabolic aging model induced by HFD	Liver, adipose tissue, pancreas	Ameliorates metabolic dysfunction	(66)
NKG2D	Senescent mouse model	Brain	Target senescent brain cells	(68)
BCMA/CD24	Human MM cell lines xenograft mouse model	Multiple myeloma	Control growth	(180)
CD5/CD7	Mouse xenograft model	T cell malignancies	Mitigate tumor antigen escape	(Dai et al. 2022)
CD33/CLL-1	U937-Luc xenograft NSG mouse model	B cell malignancies	Ttreat acute myeloid leukemia	(114)
FOLR1/MSLN	Subcutaneous SKOV3 xenograft model in B-NDG mice	Ovarian	Enhance antitumor effects	(Liang et al. 2021)
Her2	Nude mouse tumor transplantation model	Liver	Safe tumor suppression	(Xue et al. 2018)
GD3	TSC2 gene knockout tumor cell inoculated mouse model;Spontaneous Tumor Model in TSC2 Heterozygous Mice	Liver and kidney	Target tumors	(179)
CD24	5TGM1/KaLwRij MM mouse models	Myeloma	Reduce MM burden	(180)

This table summarizes representative preclinical studies investigating novel target antigens for chimeric antigen receptor T-cell (CAR-T) therapy, focusing on senolytic applications, hematological malignancies, and solid tumors..

### 5.3 Safety risks and management

Cytokine release syndrome (CRS), immune effector cell-associated neurotoxicity syndrome (ICANS), and off-target effects represent major safety risks in CAR-T therapy. Elderly patients, with reduced multi-organ functional reserve, exhibit poorer tolerance to these toxicities. CRS is the most common toxicity associated with CAR-T cell therapy, presenting symptoms ranging from mild to life-threatening (181). CRS management follows the principle of “prevention-oriented, tiered intervention, and multi-pronged approach.” Among these, targeted therapy against the IL-6 signaling axis is the current standard treatment strategy for CRS (182), effectively alleviating symptoms and improving patient prognosis. In 2019, the American Society for Transplantation and Cellular Therapy released updated guidelines establishing a unified consensus grading system for CRS and neurotoxicity (183). ICANS occurs less frequently and typically presents later than CRS. The pathophysiology and management of ICANS remain unclear, but pre-infusion immune profiling may alter susceptibility to ICANS (184). Notably, blocking the IL-6 receptor system appears ineffective for ICANS (185), whereas inhibiting myeloid mediators such as IL-1 may improve neurotoxicity in mouse models (186–188) and may hold clinical relevance (Park et al.). A deeper understanding of the immune response after CAR-T cell infusion will help improve clinical management and strengthen our research on CAR-T cell activation or depletion, reducing post-infusion risks.

Notably, the risk of CRS and ICANS is significantly higher in elderly patients receiving fourth-generation IL-12-secreting CAR-T cells (189). The pre-existing inflammaging state in elderly patients creates a synergistic effect with CAR-T-secreted IL-12, leading to exaggerated inflammatory responses. To address this specific risk, we recommend the following age-specific safety optimization strategies: (1) avoid systemic administration of IL-12-secreting CAR-T cells in elderly patients; (2) use inducible promoter systems to restrict cytokine secretion to the target tissue; (3) replace IL-12 with less pro-inflammatory cytokines such as IL-15 or IL-21; and (4) implement low-dose fractionated infusion protocols to prevent sudden cytokine surges. (126, 190, 191).

### 5.4 Manufacturing complexity and accessibility

Existing CAR-T therapies heavily rely on autologous T cells, involving complex preparation processes, extended timelines (typically 2–4 weeks), and high costs (exceeding $500,000 per treatment) (192). Additionally, elderly patients often exhibit reduced T-cell numbers and diminished quality, increasing preparation failure. To address autologous dependency, universal allogeneic CAR-T cells can be developed by knocking out TCR and CD52 genes using technologies like CRISPR/Cas9, thereby lowering graft-versus-host disease and host rejection risks (193). Such off-the-shelf products also enable large-scale manufacturing, with projected cost reductions exceeding 50%, and have already entered clinical trials for hematologic malignancies. Non-viral vector systems (e.g., Sleeping Beauty transposon or mRNA electroporation) avoid the complexity and potential risks associated with viral vectors while shortening the production cycle (194, 195). Automation has become fundamental to CAR-T cell manufacturing, streamlining processes, enhancing scalability, and mitigating challenges posed by skill shortages and high costs (196). Directly injecting LNP-mRNA or AAV (adeno-associated virus) formulations into patients to generate CAR-T cells in vivo also reduces associated costs like ex vivo manufacturing (197). Emerging preparation techniques include novel microfluidic chip systems that efficiently perform T-cell activation and gene transduction within a closed, highly controlled environment, demonstrating strong translational potential (83, 198–200).

## 6 Conclusion

Cellular senescence represents a core biological basis for the onset and progression of multiple age-related diseases. With advancing age, senescent cells accumulate within the body, not only directly impairing tissue regeneration and repair capabilities but also disrupting microenvironmental homeostasis through the secretion of a cascade of inflammatory factors (SASP), ultimately leading to organ dysfunction and disease progression. In recent years, CAR-T therapy, a technology originating from tumor immunotherapy, has emerged as a novel and promising strategy for intervening in aging-related diseases due to its ability to precisely identify and eliminate specific target cells. This article systematically explores the key molecular mechanisms of cellular senescence, including progressive telomere shortening, activation of critical pathways such as p53-p21 and p16-Rb, and the pathogenic effects of SASP. It reviews the design principles and developmental history of CAR-T technology, highlighting its application progress in aging-related tumors, neurodegenerative diseases, cardiovascular pathologies, and metabolic disorders based on the latest research findings. Research indicates that CAR-T cells can effectively eliminate pathologically aged cells by targeting surface markers such as uPAR, NKG2DLs, and FAP. Furthermore, by modulating the senescent state of T cells themselves, CAR-T therapy enhances their functional persistence and demonstrates significant therapeutic efficacy across multiple preclinical models.

However, the widespread application of CAR-T therapy for age-related diseases still faces numerous challenges. For example, elderly patients often suffer from immune senescence, leading to a decline in autologous T cell function. Therefore, strategies such as using allogeneic T cells, implementing anti-aging genetic engineering, and optimizing in vitro culture conditions are particularly important. Insufficient target specificity remains a major hurdle. Current solutions under exploration include developing dual-target CAR systems, designing CAR structures activated by microenvironmental conditions, and identifying novel targets with greater aging specificity. Regarding safety, controllable CARs (such as inducible CARs or those with built-in “suicide switches”), cytokine blockers, and personalized dosing regimens are under active investigation. Additionally, the traditional CAR-T preparation process is complex and costly, limiting its accessibility. Efforts to address this include developing universal CAR-T cells, utilizing non-viral vector technologies, and implementing automated manufacturing processes, which hold promise for expanding patient access.

Looking ahead, CAR-T therapy has yet to be applied to kidney aging-related diseases. However, as the pathogenic mechanisms of cellular senescence in chronic kidney disease, renal fibrosis, diabetic nephropathy, and other kidney aging-related conditions become increasingly clear, targeted clearance of senescent cells will emerge as a crucial strategy for intervening in the progression of these diseases. Leveraging their highly specific antigen recognition and cytotoxic capabilities, CAR-T cell therapies have demonstrated significant efficacy in organ aging models such as the liver and lung, laying a solid foundation for their application in renal diseases. In the future, with the continuous identification of kidney-specific senescence markers, CAR-T therapy holds promise for precisely eliminating senescent populations such as tubular epithelial cells and fibroblasts, blocking SASP-mediated inflammation and fibrosis processes, thereby delaying renal function decline.

Ultimately, through deepening our understanding of aging biology and continuous optimization of CAR-T technology, this approach may not only become a crucial tool for treating age-related diseases but also hold promise for extending human “healthy lifespan,” offering a powerful new weapon in the fight against aging.

## References

[1] Shreya D Fish PN Du D . Navigating the future of elderly healthcare: A comprehensive analysis of aging populations and mortality trends using national inpatient sample (NIS) data, (2010-2024). Cureus. (2025) 17:e80442. doi: 10.7759/cureus.80442 40225437 PMC11986089

[2] Zhao D Wang Y Wong ND Wang J . Impact of aging on cardiovascular diseases: From chronological observation to biological insights: JACC family series. JACC Asia. (2024) 4:345–58. doi: 10.1016/j.jacasi.2024.02.002 PMC11099824 38765662

[3] Gasek NS Kuchel GA Kirkland JL Xu M . Strategies for targeting senescent cells in human disease. Nat Aging. (2021) 1:870–79. doi: 10.1038/s43587-021-00121-8 34841261 PMC8612694

[4] He S Sharpless NE . Senescence in health and disease. Cell. (2017) 169:1000–11. doi: 10.1016/j.cell.2017.05.015 28575665 PMC5643029

[5] Sharpless NE Sherr CJ . Forging a signature of *in vivo* senescence. Nat Rev Cancer. (2015) 15:397–408. doi: 10.1038/nrc3960 26105537

[6] Ajoolabady A Pratico D Bahijri S Eldakhakhny B Tuomilehto J Wu F . Hallmarks and mechanisms of cellular senescence in aging and disease. Cell Death Discov. (2025) 11:364. doi: 10.1038/s41420-025-02655-x 40759632 PMC12322153

[7] Xu L Wang Y Wang J Zhai J Ren L Zhu G . Radiation-induced osteocyte senescence alters bone marrow mesenchymal stem cell differentiation potential via paracrine signaling. Int J Mol Sci. (2021) 22. doi: 10.3390/ijms22179323 34502232 PMC8430495

[8] Javali PS Kumar A Sarkar S Sree Varshini R Jose Mathew D Thirumurugan K . Next-gen senotherapeutics: AI/ML-driven strategies for aging and age-related disorders. Adv Pharmacol. (2025) 104:87–119. doi: 10.1016/bs.apha.2025.01.017 40716942

[9] Wang X Zhang C Su J Ren S Wang X Zhang Y . Rejuvenation strategy for inducing and enhancing autoimmune response to eliminate senescent cells. Aging Dis. (2024) 16:2273–92. doi: 10.14336/ad.2024.0579 39122450 PMC12221410

[10] Wu J Zhang L Zhao Z Liu Y Li Z Feng X . Advancing T-cell immunotherapy for cellular senescence and disease: Mechanisms, challenges, and clinical prospects. Ageing Res Rev. (2025) 109:102783. doi: 10.1016/j.arr.2025.102783 40412763

[11] Zugasti I Espinosa-Aroca L Fidyt K Mulens-Arias V Diaz-Beya M Juan M . CAR-T cell therapy for cancer: current challenges and future directions. Signal Transduct Target Ther. (2025) 10:210. doi: 10.1038/s41392-025-02269-w 40610404 PMC12229403

[12] Al-Bari AA Al Mamun A . Current advances in regulation of bone homeostasis. FASEB Bioadv. (2020) 2:668–79. doi: 10.1096/fba.2020-00058 33205007 PMC7655096

[13] d'Adda di Fagagna F . Living on a break: cellular senescence as a DNA-damage response. Nat Rev Cancer. (2008) 8:512–22. doi: 10.1038/nrc2440 18574463

[14] Vulpis E Cuollo L Borrelli C Antonangeli F Masuelli L Cippitelli M . Doxorubicin-mediated miR-433 expression on exosomes promotes bystander senescence in multiple myeloma cells in a DDR-independent manner. Int J Mol Sci. (2023) 24. doi: 10.3390/ijms24076862 37047835 PMC10095495

[15] Yang L Yuan L . The role and intrinsic connection of cellular senescence and cell death in inflammatory bowel disease. Front Cell Dev Biol. (2025) 13:1502531. doi: 10.3389/fcell.2025.1502531 40342931 PMC12058900

[16] Dogan F Forsyth NR . Telomerase regulation: a role for epigenetics. Cancers (Basel). (2021) 13. doi: 10.3390/cancers13061213 33802026 PMC8000866

[17] Mensà E Latini S Ramini D Storci G Bonafè M Olivieri F . The telomere world and aging: Analytical challenges and future perspectives. Ageing Res Rev. (2019) 50:27–42. doi: 10.1016/j.arr.2019.01.004 30615937

[18] Liu M Zhang Y Jian Y Gu L Zhang D Zhou H . The regulations of telomerase reverse transcriptase (TERT) in cancer. Cell Death Dis. (2024) 15:90. doi: 10.1038/s41419-024-06454-7 38278800 PMC10817947

[19] Zhao J Nguyen LNT Nguyen LN Dang X Cao D Khanal S . ATM deficiency accelerates DNA damage, telomere erosion, and premature T cell aging in HIV-infected individuals on antiretroviral therapy. Front Immunol. (2019) 10:2531. doi: 10.3389/fimmu.2019.02531 31781094 PMC6856652

[20] Breen EC Sehl ME Shih R Langfelder P Wang R Horvath S . Accelerated aging with HIV begins at the time of initial HIV infection. iScience. (2022) 25:104488. doi: 10.1016/j.isci.2023.107381 35880029 PMC9308149

[21] Zhu L Liu Y Wu X Ren Y Zhang Q Ren L . Cerebroprotein hydrolysate-I protects senescence-induced by D-galactose in PC12 cells and mice. Food Sci Nutr. (2021) 9:3722–31. doi: 10.1002/fsn3.2333 34262731 PMC8269606

[22] Abuetabh Y Wu HH Chai C Al Yousef H Persad S Sergi CM . DNA damage response revisited: the p53 family and its regulators provide endless cancer therapy opportunities. Exp Mol Med. (2022) 54:1658–69. doi: 10.1038/s12276-022-00863-4 36207426 PMC9636249

[23] Nelson JA Krishnamurthy J Menezes P Liu Y Hudgens MG Sharpless NE . Expression of p16(INK4a) as a biomarker of T-cell aging in HIV-infected patients prior to and during antiretroviral therapy. Aging Cell. (2012) 11:916–8. doi: 10.1111/j.1474-9726.2012.00856.x PMC3697001 22738669

[24] Kyritsi K Francis H Zhou T Ceci L Wu N Yang Z . Downregulation of p16 decreases biliary damage and liver fibrosis in the Mdr2(/) mouse model of primary sclerosing cholangitis. Gene Expr. (2020) 20:89–103. doi: 10.3727/105221620x15889714507961 32393417 PMC7650011

[25] Joruiz SM Beck JA Horikawa I Harris CC . The Δ133p53 isoforms, tuners of the p53 pathway. Cancers (Basel). (2020) 12. doi: 10.3390/cancers12113422 33218139 PMC7698932

[26] Rolt A Nair A Cox LS . Optimisation of a screening platform for determining IL-6 inflammatory signalling in the senescence-associated secretory phenotype (SASP). Biogerontology. (2019) 20:359–71. doi: 10.1007/s10522-019-09796-4 30741380 PMC6535418

[27] Acosta JC Banito A Wuestefeld T Georgilis A Janich P Morton JP . A complex secretory program orchestrated by the inflammasome controls paracrine senescence. Nat Cell Biol. (2013) 15:978–90. doi: 10.1038/ncb2784 23770676 PMC3732483

[28] Herbstein F Sapochnik M Attorresi A Pollak C Senin S Gonilski-Pacin D . The SASP factor IL-6 sustains cell-autonomous senescent cells via a cGAS-STING-NFκB intracrine senescent noncanonical pathway. Aging Cell. (2024) 23:e14258. doi: 10.1111/acel.14258 39012326 PMC11464112

[29] Odawara T Yamauchi S Ichijo H . Apoptosis signal-regulating kinase 1 promotes inflammation in senescence and aging. Commun Biol. (2024) 7:691. doi: 10.1038/s42003-024-06386-0 38839869 PMC11153534

[30] Klepacki H Kowalczuk K Łepkowska N Hermanowicz JM . Molecular regulation of SASP in cellular senescence: therapeutic implications and translational challenges. Cells. (2025) 14. doi: 10.3390/cells14130942 40643463 PMC12248485

[31] Suzuki K Susaki EA Nagaoka I . Lipopolysaccharides and cellular senescence: Involvement in atherosclerosis. Int J Mol Sci. (2022) 23. doi: 10.3390/ijms231911148 36232471 PMC9569556

[32] Salminen A Kauppinen A Kaarniranta K . Emerging role of NF-κB signaling in the induction of senescence-associated secretory phenotype (SASP). Cell Signal. (2012) 24:835–45. doi: 10.1016/j.cellsig.2011.12.006 22182507

[33] Alqahtani S Alqahtani T Venkatesan K Sivadasan D Ahmed R Sirag N . SASP modulation for cellular rejuvenation and tissue homeostasis: therapeutic strategies and molecular insights. Cells. (2025) 14. doi: 10.3390/cells14080608 40277933 PMC12025513

[34] Kale A Sharma A Stolzing A Desprez PY Campisi J . Role of immune cells in the removal of deleterious senescent cells. Immun Ageing. (2020) 17:16. doi: 10.1186/s12979-020-00187-9 32518575 PMC7271494

[35] Han Z Wang K Ding S Zhang M . Cross-talk of inflammation and cellular senescence: a new insight into the occurrence and progression of osteoarthritis. Bone Res. (2024) 12:69. doi: 10.1038/s41413-024-00375-z 39627227 PMC11615234

[36] Dou X Fu Q Long Q Liu S Zou Y Fu D . PDK4-dependent hypercatabolism and lactate production of senescent cells promotes cancer Malignancy. Nat Metab. (2023) 5:1887–910. doi: 10.1038/s42255-023-00912-w 37903887 PMC10663165

[37] Ma L Yu J Fu Y He X Ge S Jia R . The dual role of cellular senescence in human tumor progression and therapy. MedComm (2020). (2024) 5:e695. doi: 10.1002/mco2.695 39161800 PMC11331035

[38] Admasu TD Yu JS . Harnessing immune rejuvenation: advances in overcoming T cell senescence and exhaustion in cancer immunotherapy. Aging Cell. (2025) 24:e70055. doi: 10.1111/acel.70055 40178455 PMC12073907

[39] Van den Hove LE Van Gool SW Vandenberghe P Boogaerts MA Ceuppens JL . CD57+/CD28- T cells in untreated hemato-oncological patients are expanded and display a Th1-type cytokine secretion profile, ex vivo cytolytic activity and enhanced tendency to apoptosis. Leukemia. (1998) 12:1573–82. doi: 10.1038/sj.leu.2401146 9766502

[40] Liu X Mo W Ye J Li L Zhang Y Hsueh EC . Regulatory T cells trigger effector T cell DNA damage and senescence caused by metabolic competition. Nat Commun. (2018) 9:249. doi: 10.1038/s41467-017-02689-5 29339767 PMC5770447

[41] Dong Z Luo Y Yuan Z Tian Y Jin T Xu F . Cellular senescence and SASP in tumor progression and therapeutic opportunities. Mol Cancer. (2024) 23:181. doi: 10.1186/s12943-024-02096-7 39217404 PMC11365203

[42] Salvador AFM Abduljawad N Kipnis J . Meningeal lymphatics in central nervous system diseases. Annu Rev Neurosci. (2024) 47:323–44. doi: 10.1146/annurev-neuro-113023-103045 38648267 PMC12051392

[43] Rim C You MJ Nahm M Kwon MS . Emerging role of senescent microglia in brain aging-related neurodegenerative diseases. Transl Neurodegener. (2024) 13:10. doi: 10.1186/s40035-024-00402-3 38378788 PMC10877780

[44] Joers V Tansey MG Mulas G Carta AR . Microglial phenotypes in Parkinson's disease and animal models of the disease. Prog Neurobiol. (2017) 155:57–75. doi: 10.1016/j.pneurobio.2016.04.006 27107797 PMC5073045

[45] Windener F Grewing L Thomas C Dorion MF Otteken M Kular L . Physiological aging and inflammation-induced cellular senescence may contribute to oligodendroglial dysfunction in MS. Acta Neuropathol. (2024) 147:82. doi: 10.1007/s00401-024-02733-x 38722375 PMC11082024

[46] Rezagholizadeh N Datta G Hasler WA Nguon EC Smokey EV Chen X . TLR7 mediates HIV-1 tat-induced cellular senescence in human astrocytes. Aging Cell. (2025) 24:e70086. doi: 10.1111/acel.70086 40304459 PMC12266786

[47] Xu C Qiu Z Guo Q Huang Y Zhao Y Zhao R . The role of cellular senescence in cardiovascular disease. Cell Death Discov. (2025) 11:431. doi: 10.1038/s41420-025-02720-5 41053058 PMC12501035

[48] Ekun OA Fasela EO Oladele DA Liboro GO Raheem TY . Risks of cardio-vascular diseases among highly active antiretroviral therapy (HAART) treated HIV seropositive volunteers at a treatment centre in Lagos, Nigeria. Pan Afr Med J. (2021) 38:206. doi: 10.11604/pamj.2021.38.206.26791 33995812 PMC8106780

[49] Chen YF Stampley JE Irving BA Dugas TR . Chronic nucleoside reverse transcriptase inhibitors disrupt mitochondrial homeostasis and promote premature endothelial senescence. Toxicol Sci. (2019) 172:445–56. doi: 10.31390/gradschool_dissertations.4974 31545371

[50] Vidal F Domingo JC Guallar J Saumoy M Cordobilla B Sánchez de la Rosa R . *In vitro* cytotoxicity and mitochondrial toxicity of tenofovir alone and in combination with other antiretrovirals in human renal proximal tubule cells. Antimicrob Agents Chemother. (2006) 50:3824–32. doi: 10.1128/aac.00437-06 16940060 PMC1635212

[51] Smith RA Murphy MP . Mitochondria-targeted antioxidants as therapies. Discov Med. (2011) 11:106–14. doi: 10.1201/b12308-8 21356165

[52] Vellasamy DM Lee SJ Goh KW Goh BH Tang YQ Ming LC . Targeting immune senescence in atherosclerosis. Int J Mol Sci. (2022) 23. doi: 10.3390/ijms232113059 36361845 PMC9658319

[53] Wong LS van der Harst P de Boer RA Huzen J van Gilst WH van Veldhuisen DJ . Aging, telomeres and heart failure. Heart Fail Rev. (2010) 15:479–86. doi: 10.1007/s10741-010-9173-7 20532978 PMC2919688

[54] Chanvillard L Mason T Ferenbach DA . Understanding and targeting senescence in kidney disease. Clin Kidney J. (2025) 18:sfaf190. doi: 10.1093/ckj/sfaf190 40761301 PMC12319541

[55] Sato Y Yanagita M . Immunology of the ageing kidney. Nat Rev Nephrol. (2019) 15:625–40. doi: 10.1038/s41581-019-0185-9 31477915

[56] Fang Y Gong AY Haller ST Dworkin LD Liu Z Gong R . The ageing kidney: molecular mechanisms and clinical implications. Ageing Res Rev. (2020) 63:101151. doi: 10.1016/j.arr.2020.101151 32835891 PMC7595250

[57] Chen J Zhang H Yi X Dou Q Yang X He Y . Cellular senescence of renal tubular epithelial cells in acute kidney injury. Cell Death Discov. (2024) 10:62. doi: 10.1038/s41420-024-01831-9 38316761 PMC10844256

[58] Rex N Melk A Schmitt R . Cellular senescence and kidney aging. Clin Sci (Lond). (2023) 137:1805–21. doi: 10.1042/cs20230140 38126209 PMC10739085

[59] Park CH . Making potent CAR T cells using genetic engineering and synergistic agents. Cancers (Basel). (2021) 13. doi: 10.3390/cancers13133236 34209505 PMC8269169

[60] Awasthi R Maier HJ Zhang J Lim S . Kymriah® (tisagenlecleucel) - an overview of the clinical development journey of the first approved CAR-T therapy. Hum Vaccin Immunother. (2023) 19:2210046. doi: 10.1080/21645515.2023.2210046 37185251 PMC10294746

[61] Hughes-Parry HE Cross RS Jenkins MR . The evolving protein engineering in the design of chimeric antigen receptor T cells. Int J Mol Sci. (2019) 21. doi: 10.3390/ijms21010204 31892219 PMC6981602

[62] June CH Sadelain M . Chimeric antigen receptor therapy. N Engl J Med. (2018) 379:64–73. doi: 10.1056/nejmra1706169 29972754 PMC7433347

[63] Zhang C Liu J Zhong JF Zhang X . Engineering CAR-T cells. biomark Res. (2017) 5:22. doi: 10.1186/s40364-017-0102-y 28652918 PMC5482931

[64] Ma HY Das J Prendergast C De Jong D Braumuller B Paily J . Advances in CAR T cell therapy for non-small cell lung cancer. Curr Issues Mol Biol. (2023) 45:9019–38. doi: 10.3390/cimb45110566 37998743 PMC10670348

[65] Maiorana G Antolino G La Verde G Tafuri A . CAR-T therapy in multiple myeloma: looking beyond. Hemato. (2024) 5:180–98. doi: 10.3390/hemato5020015 30654563

[66] Amor C Fernández-Maestre I Chowdhury S Ho YJ Nadella S Graham C . Prophylactic and long-lasting efficacy of senolytic CAR T cells against age-related metabolic dysfunction. Nat Aging. (2024) 4:336–49. doi: 10.1038/s43587-023-00560-5 38267706 PMC10950785

[67] Amor C Feucht J Leibold J Ho YJ Zhu C Alonso-Curbelo D . Senolytic CAR T cells reverse senescence-associated pathologies. Nature. (2020) 583:127–32. doi: 10.1038/s41586-020-2403-9 32555459 PMC7583560

[68] Deng Y Kumar A Xie K Schaaf K Scifo E Morsy S . Targeting senescent cells with NKG2D-CAR T cells. Cell Death Discov. (2024) 10:217. doi: 10.1038/s41420-024-01976-7 38704364 PMC11069534

[69] Ramos CA Rouce R Robertson CS Reyna A Narala N Vyas G . *In vivo* fate and activity of second- versus third-generation CD19-specific CAR-T cells in B cell non-hodgkin's lymphomas. Mol Ther. (2018) 26:2727–37. doi: 10.1016/j.ymthe.2018.09.009 30309819 PMC6277484

[70] Chen T Wang M Chen Y Liu Y . Current challenges and therapeutic advances of CAR-T cell therapy for solid tumors. Cancer Cell Int. (2024) 24:133. doi: 10.1186/s12935-024-03315-3 38622705 PMC11017638

[71] Rosas-Campos R Arceo-Orozco S Sandoval-Rodriguez A Madrigal JA Armendariz-Borunda J . Above and beyond senescence and CAR T cell: advances and future perspectives. Front Immunol. (2025) 16:1701655. doi: 10.3389/fimmu.2025.1701655 41476987 PMC12747912

[72] Alviano AM Biondi M Grassenis E Biondi A Serafini M Tettamanti S . Fully equipped CARs to address tumor heterogeneity, enhance safety, and improve the functionality of cellular immunotherapies. Front Immunol. (2024) 15:1407992. doi: 10.3389/fimmu.2024.1407992 38887285 PMC11180895

[73] Miao L Zhang J Huang B Zhang Z Wang S Tang F . Special chimeric antigen receptor (CAR) modifications of T cells: A review. Front Oncol. (2022) 12:832765. doi: 10.3389/fonc.2022.832765 35392217 PMC8981721

[74] Rallis KS Hillyar CRT Sideris M Davies JK . T-cell-based immunotherapies for haematological cancers, part B: A SWOT analysis of adoptive cell therapies. Anticancer Res. (2021) 41:1143–56. doi: 10.21873/anticanres.14871 33788705

[75] Asmamaw Dejenie T Medhin M. Tiruneh G Dessie Terefe G Tadele Admasu F Wale Tesega W Chekol Abebe E . Current updates on generations, approvals, and clinical trials of CAR T-cell therapy. Hum Vaccin Immunother. (2022) 18:2114254. doi: 10.1080/21645515.2022.2114254 36094837 PMC9746433

[76] Chan JD Scheffler CM Munoz I Sek K Lee JN Huang YK . FOXO1 enhances CAR T cell stemness, metabolic fitness and efficacy. Nature. (2024) 629:201–10. doi: 10.1038/s41586-024-07242-1 38600376 PMC11062918

[77] Can I Siegler EL Sirpilla OL Manriquez-Roman C Yun K Stewart CM . Differential susceptibility and role for senescence in CART cells based on costimulatory domains. Mol Cancer. (2025) 24:172. doi: 10.1186/s12943-025-02371-1 40495168 PMC12150488

[78] Lu J Jiang G . The journey of CAR-T therapy in hematological Malignancies. Mol Cancer. (2022) 21:194. doi: 10.1186/s12943-022-01663-0 36209106 PMC9547409

[79] Xu Z Wang R Xu Y Qiu R Chen J Liu L . Comparative analysis and process optimization for manufacturing CAR-T using the PiggyBac system derived from cryopreserved versus fresh PBMCs. Sci Rep. (2025) 15:5023. doi: 10.1038/s41598-025-89686-7 39934258 PMC11814250

[80] Hermans D Gautam S García-Cañaveras JC Gromer D Mitra S Spolski R . Lactate dehydrogenase inhibition synergizes with IL-21 to promote CD8(+) T cell stemness and antitumor immunity. Proc Natl Acad Sci USA. (2020) 117:6047–55. doi: 10.1073/pnas.1920413117 32123114 PMC7084161

[81] Zou B Wang M Bai S Li N Fan Z Peng Y . Novel mRNA-engineered fully human CAR-T cells targeting AXL in solid tumors. Biomedicines. (2025) 13. doi: 10.3390/biomedicines13040844 40299452 PMC12024984

[82] Panjwani MK Smith JB Schutsky K Gnanandarajah J O'Connor CM Powell DJ . Feasibility and safety of RNA-transfected CD20-specific chimeric antigen receptor T cells in dogs with spontaneous B cell lymphoma. Mol Ther. (2016) 24:1602–14. doi: 10.1038/mt.2016.146 27401141 PMC5113111

[83] Sytsma BJ Allain V Bourke S Faizee F Fathi M Ferreira LMR . Scalable intracellular delivery via microfluidic vortex shedding enhances the function of chimeric antigen receptor T-cells. Sci Rep. (2025) 15:5749. doi: 10.1038/s41598-025-89070-5 39962112 PMC11832915

[84] Zhang Z Ma B Li B Li Z Gao M Zhao H . Cardiolipin-mimic lipid nanoparticles without antibody modification delivered senolytic *in vivo* CAR-T therapy for inflamm-aging. Cell Rep Med. (2025) 6:102209. doi: 10.1016/j.xcrm.2025.102209 40602406 PMC12281384

[85] Porter DL Levine BL Kalos M Bagg A June CH . Chimeric antigen receptor-modified T cells in chronic lymphoid leukemia. N Engl J Med. (2011) 365:725–33. doi: 10.1056/nejmoa1103849 21830940 PMC3387277

[86] Goyco Vera D Waghela H Nuh M Pan J Lulla P . Approved CAR-T therapies have reproducible efficacy and safety in clinical practice. Hum Vaccin Immunother. (2024) 20:2378543. doi: 10.1080/21645515.2024.2378543 39104200 PMC11305028

[87] Anderson LD . Idecabtagene vicleucel (ide-cel) CAR T-cell therapy for relapsed and refractory multiple myeloma. Future Oncol. (2022) 18:277–89. doi: 10.2217/fon-2021-1090 34854741

[88] Maude SL Laetsch TW Buechner J Rives S Boyer M Bittencourt H . Tisagenlecleucel in children and young adults with B-cell lymphoblastic leukemia. N Engl J Med. (2018) 378:439–48. doi: 10.1056/nejmoa1709866 29385370 PMC5996391

[89] Zhu H Deng H Mu J Lyu C Jiang Y Deng Q . Anti-CD22 CAR-T cell therapy as a salvage treatment in B cell Malignancies refractory or relapsed after anti-CD19 CAR-T therapy. Onco Targets Ther. (2021) 14:4023–37. doi: 10.2147/ott.s312904 34239307 PMC8259947

[90] Dong Y Yang T Zhao M Song F Chen R Zhang M . Safety and efficacy of autologous humanized CD19 CAR-T cell therapy for relapsed/refractory B-cell non-Hodgkin lymphoma. Bone Marrow Transplant. (2025) 60:1445–50. doi: 10.1038/s41409-025-02691-2 40836026 PMC12583143

[91] Xue SL Liu MJ Qian CS Chen SN Qiu HY Kang LQ . IL-6 knockdown anti-CD19 CAR-T cells (ssCART-19) for patients with relapsed or refractory acute lymphoblastic leukemia: Phase 1 trial. Blood Cancer J. (2025) 15:182. doi: 10.1038/s41408-025-01397-4 41145445 PMC12559308

[92] Nie T . Talicabtagene autoleucel: first approval. Mol Diagn Ther. (2024) 28:495–9. doi: 10.1007/s40291-024-00719-9 38780683

[93] Si X Shao M Teng X Huang Y Meng Y Wu L . Mitochondrial isocitrate dehydrogenase impedes CAR T cell function by restraining antioxidant metabolism and histone acetylation. Cell Metab. (2024) 36:176–192.e10. doi: 10.1016/j.cmet.2023.12.010 38171332

[94] Patel KK Tariveranmoshabad M Kadu S Shobaki N June C . From concept to cure: The evolution of CAR-T cell therapy. Mol Ther. (2025) 33:2123–40. doi: 10.1016/j.ymthe.2025.03.005 40070120 PMC12126787

[95] Zhao B Wu B Feng N Zhang X Zhang X Wei Y . Aging microenvironment and antitumor immunity for geriatric oncology: The landscape and future implications. J Hematol Oncol. (2023) 16:28. doi: 10.1186/s13045-023-01426-4 36945046 PMC10032017

[96] Abdalsalam NMF Ibrahim A Saliu MA Liu TM Wan X Yan D . MDSC: a new potential breakthrough in CAR-T therapy for solid tumors. Cell Commun Signal. (2024) 22:612. doi: 10.1186/s12964-024-01995-y 39702149 PMC11660884

[97] Liu Q Li J Sun X Lin J Yu Z Xiao Y . Immunosenescence and cancer: molecular hallmarks, tumor microenvironment remodeling, and age-specific immunotherapy challenges. J Hematol Oncol. (2025) 18:81. doi: 10.1186/s13045-025-01735-w 40846970 PMC12374445

[98] Mehta PH Trollope GS Leung P Chinni SS Iasinskaia A Harrison AJ . Choice of activation protocol impacts the yield and quality of CAR T cell product, particularly with older individuals. Clin Transl Immunol. (2024) 13:e70016. doi: 10.1002/cti2.70016 39619015 PMC11605362

[99] Park JA Cheung NV . Overcoming tumor heterogeneity by ex vivo arming of T cells using multiple bispecific antibodies. J Immunother Cancer. (2022) 10. doi: 10.1136/jitc-2021-003771 35086947 PMC8796264

[100] Zhang B Wu J Jiang H Zhou M . Strategies to overcome antigen heterogeneity in CAR-T cell therapy. Cells. (2025) 14. doi: 10.3390/cells14050320 40072049 PMC11899321

[101] Kobayashi A Nobili A Neier SC Sadiki A Distel R Zhou ZS . Light-controllable binary switch activation of CAR T cells. ChemMedChem. (2022) 17:e202100722. doi: 10.1002/cmdc.202100722 35146940 PMC9304291

[102] Dercle L McGale J Sun S Marabelle A Yeh R Deutsch E . Artificial intelligence and radiomics: fundamentals, applications, and challenges in immunotherapy. J Immunother Cancer. (2022) 10. doi: 10.1136/jitc-2022-005292 36180071 PMC9528623

[103] Piraner DI Abedi MH Duran Gonzalez MJ Chazin-Gray A Lin A Zhu I . Engineered receptors for soluble cellular communication and disease sensing. Nature. (2025) 638:805–13. doi: 10.1038/s41586-024-08366-0 39542025 PMC11839477

[104] Murad JP Christian L Rosa R Ren Y Buckley AJ Lee EHJ . Solid tumour CAR-T cells engineered with fusion proteins targeting PD-L1 for localized IL-12 delivery. Nat BioMed Eng. (2025). doi: 10.1038/s41551-025-01509-2 41034514 PMC13099387

[105] Xu J Ding L Mei J Hu Y Kong X Dai S . Dual roles and therapeutic targeting of tumor-associated macrophages in tumor microenvironments. Signal Transduct Target Ther. (2025) 10:268. doi: 10.1038/s41392-025-02325-5 40850976 PMC12375796

[106] Liu Z Zhou Z Dang Q Xu H Lv J Li H . Immunosuppression in tumor immune microenvironment and its optimization from CAR-T cell therapy. Theranostics. (2022) 12:6273–90. doi: 10.7150/thno.76854 36168626 PMC9475465

[107] Dai Z Lin X Wang X Zou X Yan Y Wang R . Ectopic CXCR2 expression cells improve the anti-tumor efficiency of CAR-T cells and remodel the immune microenvironment of pancreatic ductal adenocarcinoma. Cancer Immunol Immunother. (2024) 73:61. doi: 10.1007/s00262-024-03648-y 38430267 PMC10908625

[108] Wang Z Li P Zeng X Guo J Zhang C Fan Z . CAR-T therapy dilemma and innovative design strategies for next generation. Cell Death Dis. (2025) 16:211. doi: 10.1038/s41419-025-07454-x 40148310 PMC11950394

[109] Maalej KM Merhi M Inchakalody VP Mestiri S Alam M Maccalli C . CAR-cell therapy in the era of solid tumor treatment: current challenges and emerging therapeutic advances. Mol Cancer. (2023) 22:20. doi: 10.1186/s12943-023-01723-z 36717905 PMC9885707

[110] Roybal KT Williams JZ Morsut L Rupp LJ Kolinko I Choe JH . Engineering T cells with customized therapeutic response programs using synthetic notch receptors. Cell. (2016) 167:419–432.e16. doi: 10.1016/j.cell.2016.09.011 27693353 PMC5072533

[111] Simic MS Watchmaker PB Gupta S Wang Y Sagan SA Duecker J . Programming tissue-sensing T cells that deliver therapies to the brain. Science. (2024) 386:eadl4237. doi: 10.1126/science.adl4237 39636984 PMC11900893

[112] Zhu I Liu R Garcia JM Hyrenius-Wittsten A Piraner DI Alavi J . Modular design of synthetic receptors for programmed gene regulation in cell therapies. Cell. (2022) 185:1431–1443.e16. doi: 10.1016/j.cell.2022.03.023 35427499 PMC9108009

[113] Mazzi MT Hajdu KL Ribeiro PR Bonamino MH . CAR-T cells leave the comfort zone: current and future applications beyond cancer. Immunother Adv. (2021) 1:ltaa006. doi: 10.1093/immadv/ltaa006 36284896 PMC9585679

[114] Wang Q Xiao ZX Zheng X Wang G Yang L Shi L . *In vivo* CD19 CAR T-cell therapy for refractory systemic lupus erythematosus. N Engl J Med. (2025) 393:1542–4. doi: 10.1056/nejmc2509522 40961420

[115] Wang X Zhang Y Wang H Wu X He C Lin S . Allogeneic CD19-targeting T cells for treatment-refractory systemic lupus erythematosus: A phase 1 trial. Nat Med. (2025). doi: 10.1038/s41591-025-03899-x 40866583

[116] Li Y Li S Zhao X Sheng J Xue L Schett G . Fourth-generation chimeric antigen receptor T-cell therapy is tolerable and efficacious in treatment-resistant rheumatoid arthritis. Cell Res. (2025) 35:220–3. doi: 10.1038/s41422-024-01068-2 39779933 PMC11909189

[117] Rangel-Peláez C Martínez-Gutiérrez L Tristán-Manzano M Callejas JL Ortego-Centeno N Martín F . CD19 CAR-T cell therapy: a new dawn for autoimmune rheumatic diseases? Front Immunol. (2024) 15:1502712. doi: 10.3389/fimmu.2024.1502712 39742256 PMC11685126

[118] Herzig E Kim KC Packard TA Vardi N Schwarzer R Gramatica A . Attacking latent HIV with convertibleCAR-T cells, a highly adaptable killing platform. Cell. (2019) 179:880–894.e10. doi: 10.1016/j.cell.2019.10.002 31668804 PMC6922308

[119] Liu B Zhang W Xia B Jing S Du Y Zou F . Broadly neutralizing antibody-derived CAR T cells reduce viral reservoir in individuals infected with HIV-1. J Clin Invest. (2021) 131. doi: 10.1172/jci150211 34375315 PMC8483761

[120] Du K Umbaugh DS Wang L Jun JH Dutta RK Oh SH . Targeting senescent hepatocytes for treatment of metabolic dysfunction-associated steatotic liver disease and multi-organ dysfunction. Nat Commun. (2025) 16:3038. doi: 10.1038/s41467-025-57616-w 40155379 PMC11953480

[121] Li H Yin L Wang Y Wang X Shi M Cao J . Safety and efficacy of chimeric antigen receptor T-cell therapy in relapsed/refractory multiple myeloma with renal impairment. Bone Marrow Transplant. (2020) 55:2215–8. doi: 10.1038/s41409-020-0930-5 32388534

[122] Sidana S Peres LC Hashmi H Hosoya H Ferreri C Khouri J . Idecabtagene vicleucel chimeric antigen receptor T-cell therapy for relapsed/refractory multiple myeloma with renal impairment. Haematologica. (2024) 109:777–86. doi: 10.3324/haematol.2023.283940 37731379 PMC10905101

[123] Park H Mugundu GM Singh AP . Mechanistic evaluation of anti-CD19 CAR-T cell therapy repurposed in systemic lupus erythematosus using a quantitative systems pharmacology model. Clin Transl Sci. (2025) 18:e70146. doi: 10.5124/jkma.2009.52.7.645 39936636 PMC11815715

[124] Mackensen A Müller F Mougiakakos D Böltz S Wilhelm A Aigner M . Anti-CD19 CAR T cell therapy for refractory systemic lupus erythematosus. Nat Med. (2022) 28:2124–32. doi: 10.1038/s41591-022-02017-5 36109639

[125] Zhao S Li R Xia Y Wang X Liu Z Chu Q . Targeting ECM-producing cells with CAR-T therapy alleviates fibrosis in chronic kidney disease. Cell Stem Cell. (2025) 32:1390–1402.e9. doi: 10.1016/j.stem.2025.07.014 40848726

[126] Yang D Sun B Li S Wei W Liu X Cui X . NKG2D-CAR T cells eliminate senescent cells in aged mice and nonhuman primates. Sci Transl Med. (2023) 15:eadd1951. doi: 10.1126/scitranslmed.add1951 37585504

[127] Lian J Yue Y Yu W Zhang Y . Immunosenescence: a key player in cancer development. J Hematol Oncol. (2020) 13:151. doi: 10.1186/s13045-020-00986-z 33168037 PMC7653700

[128] Noll JH Levine BL June CH Fraietta JA . Beyond youth: Understanding CAR T cell fitness in the context of immunological aging. Semin Immunol. (2023) 70:101840. doi: 10.1016/j.smim.2023.101840 37729825

[129] Roselle C Horikawa I Chen L Kelly AR Gonzales D Da T . Enhancing chimeric antigen receptor T cell therapy by modulating the p53 signaling network with Δ133p53α. Proc Natl Acad Sci USA. (2024) 121:e2317735121. doi: 10.1073/pnas.2317735121 38408246 PMC10927528

[130] Sueangoen N Prasongtanakij S . Emerging CAR immunotherapies: Broadening therapeutic horizons beyond cancer. Clin Exp Med. (2025) 25:274. doi: 10.1007/s10238-025-01820-x 40758198 PMC12321946

[131] Klabukov I Kabakov AE Yakimova A Baranovskii D Sosin D Atiakshin D . Tumor-associated extracellular matrix obstacles for CAR-T cell therapy: approaches to overcoming. Curr Oncol. (2025) 32. doi: 10.3390/curroncol32020079 39996879 PMC11854105

[132] Bughda R Dimou P D'Souza RR Klampatsa A . Fibroblast activation protein (FAP)-targeted CAR-T cells: launching an attack on tumor stroma. Immunotargets Ther. (2021) 10:313–23. doi: 10.2147/itt.s291767 34386436 PMC8354246

[133] Ruan G Wang X Ou H Guo D . Cancer-associated fibroblasts: dual roles from senescence sentinels to death regulators and new dimensions in therapy. Front Immunol. (2025) 16:1635771. doi: 10.3389/fimmu.2025.1635771 40755775 PMC12313499

[134] Moosavi SG Rahiman N Jaafari MR Arabi L . Lipid nanoparticle (LNP) mediated mRNA delivery in neurodegenerative diseases. J Control Release. (2025) 381:113641. doi: 10.1016/j.jconrel.2025.113641 40120689

[135] Soraci L Corsonello A Paparazzo E Montesanto A Piacenza F Olivieri F . Neuroinflammaging: A tight line between normal aging and age-related neurodegenerative disorders. Aging Dis. (2024) 15:1726–47. doi: 10.14336/AD.2023.1001 PMC11272206 38300639

[136] Yoo HJ Kwon MS . Aged microglia in neurodegenerative diseases: Microglia lifespan and culture methods. Front Aging Neurosci. (2021) 13:766267. doi: 10.3389/fnagi.2021.766267 35069173 PMC8766407

[137] Cummings J . Anti-amyloid monoclonal antibodies are transformative treatments that redefine Alzheimer's disease therapeutics. Drugs. (2023) 83:569–76. doi: 10.1007/s40265-023-01858-9 37060386 PMC10195708

[138] Krishnamurthy PK Deng Y Sigurdsson EM . Mechanistic studies of antibody-mediated clearance of tau aggregates using an ex vivo brain slice model. Front Psychiatry. (2011) 2:59. doi: 10.3389/fpsyt.2011.00059 22025915 PMC3198029

[139] Fischbach F Richter J Pfeffer LK Fehse B Berger SC Reinhardt S . CD19-targeted chimeric antigen receptor T cell therapy in two patients with multiple sclerosis. Med. (2024) 5:550–58.e2. doi: 10.1016/j.medj.2024.03.002 38554710

[140] Qin C Dong MH Zhou LQ Chu YH Pang XW He JY . Anti-BCMA CAR-T therapy in patients with progressive multiple sclerosis. Cell. (2025). doi: 10.1016/j.cell.2025.09.020 41101309

[141] Zha Y Zhuang W Yang Y Zhou Y Li H Liang J . Senescence in vascular smooth muscle cells and atherosclerosis. Front Cardiovasc Med. (2022) 9:910580. doi: 10.3389/fcvm.2022.910580 35722104 PMC9198250

[142] Wong JJ Hong R Teo LLY Tan R-S Koh AS . Atherosclerotic cardiovascular disease in aging and the role of advanced cardiovascular imaging. NPJ Cardiovasc Health. (2024) 1:11. doi: 10.1038/s44325-024-00012-y 41775948 PMC12912418

[143] Liu D Liu J Zhang D Yang W . Advances in relationship between cell senescence and atherosclerosis. Zhejiang Da Xue Xue Bao Yi Xue Ban. (2022) 51:95–101. doi: 10.3724/zdxbyxb-2021-0270 35576118 PMC9109755

[144] Mondal AR Misra A . Emerging cell-specific therapies in cardiovascular disease. Vascul Pharmacol. (2025) 160:107516. doi: 10.1016/j.vph.2025.107516 40545185

[145] Luan Y Zhu X Jiao Y Liu H Huang Z Pei J . Cardiac cell senescence: molecular mechanisms, key proteins and therapeutic targets. Cell Death Discov. (2024) 10:78. doi: 10.1038/s41420-023-01792-5 38355681 PMC10866973

[146] Weissenböck V Weber L Schlederer M Silva Sousa L Stampfer A Baydar S . Molecular imaging of fibroblast activation protein in response to cardiac injury using [(68)Ga]Ga-DATA(5m).SA.FAPi. Pharm (Basel). (2025) 18. doi: 10.1155/cdr/7230505 PMC12115071 40430477

[147] Li H Zheng Q Jiang Y Yang L Li S Yang P . Fibroblast activation protein-targeted CAR-T cells induce apoptosis in murine cardiac myofibroblasts. Cardiovasc Ther. (2025) 2025:7230505. doi: 10.1155/cdr/7230505 40977952 PMC12450108

[148] Aghajanian H Kimura T Rurik JG Hancock AS Leibowitz MS Li L . Targeting cardiac fibrosis with engineered T cells. Nature. (2019) 573:430–33. doi: 10.1038/s41586-019-1546-z 31511695 PMC6752964

[149] Rurik JG Tombácz I Yadegari A Méndez Fernández PO Shewale SV Li L . CAR T cells produced *in vivo* to treat cardiac injury. Science. (2022) 375:91–6. doi: 10.9734/bpi/mono/978-81-19039-63-0/ch19 PMC9983611 34990237

[150] Liuzzo G Patrono C . *In vivo* generated chimeric antigen receptor T cells reduce fibrosis and restore cardiac function in experimental heart failure. Eur Heart J. (2022) 43:1531–2. doi: 10.1093/eurheartj/ehac090 35187570

[151] Prasad K Cross RS Jenkins MR . Progress in the development of cytokine armoured CAR T cells. Nat Rev Immunol. (2026). doi: 10.1038/s41577-026-01280-8 41792262

[152] Williams SN Ding WX . The impact of aging on liver health and the development of liver diseases. Hepatol Commun. (2025) 9. doi: 10.1097/hc9.0000000000000808 40982225 PMC12456491

[153] Mohammadi V Maleki AJ Nazari M Siahmansouri A Moradi A Elahi R . Chimeric antigen receptor (CAR)-based cell therapy for type 1 diabetes mellitus (T1DM); current progress and future approaches. Stem Cell Rev Rep. (2024) 20:585–600. doi: 10.1007/s12015-023-10668-1 38153634

[154] Wiering L Subramanian P Hammerich L . Hepatic stellate cells: Dictating outcome in nonalcoholic fatty liver disease. Cell Mol Gastroenterol Hepatol. (2023) 15:1277–92. doi: 10.1016/j.jcmgh.2023.02.010 36828280 PMC10148161

[155] Xiong H Guo J . Targeting hepatic stellate cells for the prevention and treatment of liver cirrhosis and hepatocellular carcinoma: Strategies and clinical translation. Pharm (Basel). (2025) 18. doi: 10.3390/ph18040507 40283943 PMC12030350

[156] Yashaswini CN Cogliati B Qin T To T Williamson T Papp TE . Anti-FAP CAR T cells produced in vivo reduce fibrosis and restore liver homeostasis in metabolic dysfunction-associated steatohepatitis. Sci Transl Med. (2026) 18(833):eadx0368. doi: 10.1126/scitranslmed.adx0368 41564158

[157] Yang Z Ha B Wu Q Ren F Yin Z Zhang H . Expanding the horizon of CAR T cell therapy: From cancer treatment to autoimmune diseases and beyond. Front Immunol. (2025) 16:1544532. doi: 10.3389/fimmu.2025.1544532 40046061 PMC11880241

[158] Maude SL Frey N Shaw PA Aplenc R Barrett DM Bunin NJ . Chimeric antigen receptor T cells for sustained remissions in leukemia. N Engl J Med. (2014) 371:1507–17. doi: 10.1056/nejmoa1407222 25317870 PMC4267531

[159] Chen ACY Jaiswal S Martinez D Yerinde C Ji K Miranda V . The aged tumor microenvironment limits T cell control of cancer. Nat Immunol. (2024) 25:1033–45. doi: 10.1038/s41590-024-01828-7 38745085 PMC11500459

[160] Klepin HD Rao AV Pardee TS . Acute myeloid leukemia and myelodysplastic syndromes in older adults. J Clin Oncol. (2014) 32:2541–52. doi: 10.1200/jco.2014.55.1564 25071138 PMC4876337

[161] Sobrini-Morillo P Corral-Tuesta C Sánchez-Castellano C Gutiérrez-Blanco T Palomo-Rumschisky P Álvarez-Pinheiro CG . Comprehensive geriatric assessment of older patients with multiple myeloma: A prospective observational study. Cancers (Basel). (2025) 17. doi: 10.3390/cancers17172904 40941001 PMC12428207

[162] Neelapu SS Jacobson CA Oluwole OO Munoz J Deol A Miklos DB . Outcomes of older patients in ZUMA-1, a pivotal study of axicabtagene ciloleucel in refractory large B-cell lymphoma. Blood. (2020) 135:2106–9. doi: 10.1182/blood.2019004162 32181801 PMC7273828

[163] Schuster SJ Bishop MR Tam CS Waller EK Borchmann P McGuirk JP . Tisagenlecleucel in adult relapsed or refractory diffuse large B-cell lymphoma. N Engl J Med. (2019) 380:45–56. doi: 10.1056/nejmoa1804980 30501490

[164] Munshi NC Anderson LD Shah N Madduri D Berdeja J Lonial S . Idecabtagene vicleucel in relapsed and refractory multiple myeloma. N Engl J Med. (2021) 384:705–16. doi: 10.1056/nejmoa2024850 33626253

[165] Mejia Saldarriaga M Pan D Unkenholz C Mouhieddine TH Velez-Hernandez JE Engles K . Absolute lymphocyte count after BCMA CAR-T therapy is a predictor of response and outcomes in relapsed multiple myeloma. Blood Adv. (2024) 8:3859–69. doi: 10.1182/bloodadvances.2023012470 38776397 PMC11321283

[166] Hope HC de Sostoa J Ginefra P Andreatta M Chiang YH Ronet C . Age-associated nicotinamide adenine dinucleotide decline drives CAR-T cell failure. Nat Cancer. (2025) 6:1524–36. doi: 10.1038/s43018-025-00982-7 40394194 PMC12463664

[167] Lickefett B Chu L Ortiz-Maldonado V Warmuth L Barba P Doglio M . Lymphodepletion - an essential but undervalued part of the chimeric antigen receptor T-cell therapy cycle. Front Immunol. (2023) 14:1303935. doi: 10.3389/fimmu.2023.1303935 38187393 PMC10770848

[168] Kadyrzhanova G Tamai M Sarkar S Kalra RS Ishikawa H . Aging impairs CD8 T cell responses in adoptive T-cell therapy against solid tumors. Front Immunol. (2025) 16:1484303. doi: 10.3389/fimmu.2025.1484303 39925817 PMC11803149

[169] Ullrich F Bröckelmann PJ Turki AT Khan AM Chiru ED Vetter M . Impact of immunological aging on T cell-mediated therapies in older adults with multiple myeloma and lymphoma. J Immunother Cancer. (2024) 12. doi: 10.1136/jitc-2024-009462 39622581 PMC11624774

[170] Kasakovski D Xu L Li Y . T cell senescence and CAR-T cell exhaustion in hematological Malignancies. J Hematol Oncol. (2018) 11:91. doi: 10.1186/s13045-018-0629-x 29973238 PMC6032767

[171] Rizk M Aziz J Shorr R Allan DS . Cell-based therapy using umbilical cord blood for novel indications in regenerative therapy and immune modulation: an updated systematic scoping review of the literature. Biol Blood Marrow Transplant. (2017) 23:1607–13. doi: 10.1016/j.bbmt.2017.05.032 28602892

[172] Damien P Allan DS . Regenerative therapy and immune modulation using umbilical cord blood-derived cells. Biol Blood Marrow Transplant. (2015) 21:1545–54. doi: 10.1016/j.bbmt.2015.05.022 26079441

[173] Chiao YA Zhang H Sweetwyne M Whitson J Ting YS Basisty N . Late-life restoration of mitochondrial function reverses cardiac dysfunction in old mice. Elife. (2020) 9. doi: 10.7554/elife.55513 32648542 PMC7377906

[174] Al-Chami E Tormo A Pasquin S Kanjarawi R Ziouani S Rafei M . Interleukin-21 administration to aged mice rejuvenates their peripheral T-cell pool by triggering de novo thymopoiesis. Aging Cell. (2016) 15:349–60. doi: 10.1111/acel.12440 26762709 PMC4783337

[175] Tormo A Khodayarian F Cui Y Al-Chami E Kanjarawi R Noé B . Interleukin-21 promotes thymopoiesis recovery following hematopoietic stem cell transplantation. J Hematol Oncol. (2017) 10:120. doi: 10.1186/s13045-017-0490-3 28615039 PMC5471903

[176] Gómez-Melero S Hassouneh F Vallejo-Bermúdez IM Agüera-Morales E Solana R Caballero-Villarraso J . Tandem CAR-T cell therapy: recent advances and current challenges. Front Immunol. (2025) 16:1546172. doi: 10.3389/fimmu.2025.1546172 40092990 PMC11907001

[177] Park HB Kim KH Kim JH Kim SI Oh YM Kang M . Improved safety of chimeric antigen receptor T cells indirectly targeting antigens via switchable adapters. Nat Commun. (2024) 15:9917. doi: 10.1038/s41467-024-53996-7 39557825 PMC11574259

[178] Iltis C Moskalevska I Debiesse A Seguin L Fissoun C Cervera L . A ganglioside-based immune checkpoint enables senescent cells to evade immunosurveillance during aging. Nat Aging. (2025) 5:219–36. doi: 10.1038/s43587-024-00776-z 39730825 PMC11839482

[179] Thomas A Sumughan S Dellacecca ER Shivde RS Lancki N Mukhatayev Z . Benign tumors in TSC are amenable to treatment by GD3 CAR T cells in mice. JCI Insight. (2021) 6. doi: 10.1172/jci.insight.152014 34806651 PMC8663788

[180] Sun F Cheng Y Wanchai V Guo W Mery D Xu H . Bispecific BCMA/CD24 CAR-T cells control multiple myeloma growth. Nat Commun. (2024) 15:615. doi: 10.1038/s41467-024-44873-4 38242888 PMC10798961

[181] Hay KA Hanafi LA Li D Gust J Liles WC Wurfel MM . Kinetics and biomarkers of severe cytokine release syndrome after CD19 chimeric antigen receptor-modified T-cell therapy. Blood. (2017) 130:2295–306. doi: 10.1182/blood-2017-06-793141 28924019 PMC5701525

[182] Frigault MJ Maziarz RT Park JH Lazaryan A Shah NN Svoboda J . Itacitinib for the prevention of IEC therapy-associated CRS: results from the 2-part phase 2 INCB 39110-211 study. Blood. (2025) 146:422–36. doi: 10.1182/blood.2024026586 40090005

[183] Lee DW Santomasso BD Locke FL Ghobadi A Turtle CJ Brudno JN . ASTCT consensus grading for cytokine release syndrome and neurologic toxicity associated with immune effector cells. Biol Blood Marrow Transplant. (2019) 25:625–38. doi: 10.1016/j.bbmt.2018.12.758 30592986 PMC12180426

[184] De Matteis S Dicataldo M Casadei B Storci G Laprovitera N Arpinati M . Peripheral blood cellular profile at pre-lymphodepletion is associated with CD19-targeted CAR-T cell-associated neurotoxicity. Front Immunol. (2022) 13:1058126. doi: 10.3389/fimmu.2022.1058126 36726971 PMC9886226

[185] Chen F Teachey DT Pequignot E Frey N Porter D Maude SL . Measuring IL-6 and sIL-6R in serum from patients treated with tocilizumab and/or siltuximab following CAR T cell therapy. J Immunol Methods. (2016) 434:1–8. doi: 10.1016/j.jim.2016.03.005 27049586 PMC5490247

[186] Sterner RM Sakemura R Cox MJ Yang N Khadka RH Forsman CL . GM-CSF inhibition reduces cytokine release syndrome and neuroinflammation but enhances CAR-T cell function in xenografts. Blood. (2019) 133:697–709. doi: 10.1182/blood-2018-10-881722 30463995 PMC6376281

[187] Norelli M Camisa B Barbiera G Falcone L Purevdorj A Genua M . Monocyte-derived IL-1 and IL-6 are differentially required for cytokine-release syndrome and neurotoxicity due to CAR T cells. Nat Med. (2018) 24:739–48. doi: 10.1038/s41591-018-0036-4 29808007

[188] Giavridis T van der Stegen SJC Eyquem J Hamieh M Piersigilli A Sadelain M . CAR T cell-induced cytokine release syndrome is mediated by macrophages and abated by IL-1 blockade. Nat Med. (2018) 24:731–38. doi: 10.1038/s41591-018-0041-7 29808005 PMC6410714

[189] Hawkins ER D'Souza RR Klampatsa A . Armored CAR T-cells: the next chapter in T-cell cancer immunotherapy. Biologics. (2021) 15:95–105. doi: 10.2147/btt.s291768 33883875 PMC8053711

[190] Wu Y Li YR . Frontiers of cytokine engineering in CAR cell therapy for cancer. Front Oncol. (2025) 15:1642022. doi: 10.3389/fonc.2025.1642022 41584600 PMC12827175

[191] Frigault M Rotte A Ansari A Gliner B Heery C Shah B . Dose fractionation of CAR-T cells. A systematic review of clinical outcomes. J Exp Clin Cancer Res. (2023) 42:11. doi: 10.1186/s13046-022-02540-w 36627710 PMC9830795

[192] Pinto E Lione L Compagnone M Paccagnella M Salvatori E Greco M . From ex vivo to *in vivo* chimeric antigen T cells manufacturing: new horizons for CAR T-cell based therapy. J Transl Med. (2025) 23:10. doi: 10.1186/s12967-024-06052-3 39755643 PMC11700462

[193] Wang YW Tang YM . Advances in the application strategies of CRISPR/Cas9 technology in chimeric antigen receptor T cell therapy for hematological Malignancies. Zhonghua Xue Ye Xue Za Zhi. (2025) 46:481–8. doi: 10.3760/cma.j.cn121090-20240911-00343 PMC12268297 40623912

[194] Lock D Monjezi R Brandes C Bates S Lennartz S Teppert K . Automated, scaled, transposon-based production of CAR T cells. J Immunother Cancer. (2022) 10. doi: 10.1136/jitc-2022-005189 36096530 PMC9472140

[195] Zhang J Hu Y Yang J Li W Zhang M Wang Q . Non-viral, specifically targeted CAR-T cells achieve high safety and efficacy in B-NHL. Nature. (2022) 609:369–74. doi: 10.1038/s41586-022-05140-y 36045296 PMC9452296

[196] Dias J Garcia J Agliardi G Roddie C . CAR-T cell manufacturing landscape-lessons from the past decade and considerations for early clinical development. Mol Ther Methods Clin Dev. (2024) 32:101250. doi: 10.1016/j.omtm.2024.101250 38737799 PMC11088187

[197] Bimbo JF van Diest E Murphy DE Ashoti A Evers MJW Narayanavari SA . T cell-specific non-viral DNA delivery and *in vivo* CAR-T generation using targeted lipid nanoparticles. J Immunother Cancer. (2025) 13. doi: 10.1136/jitc-2025-011759 40659448 PMC12258353

[198] Markelov V Arabuli KV Gaponenko I Sergeev V Shakirova A Lepik KV . Scalable and ultrafast CAR-T cell production using microfluidics. Lab Chip. (2025) 25:3005–15. doi: 10.1039/d5lc00139k 40396473

[199] Sin WX Jagannathan NS Teo DBL Kairi F Fong SY Tan JHL . A high-density microfluidic bioreactor for the automated manufacturing of CAR T cells. Nat BioMed Eng. (2024) 8:1571–91. doi: 10.1038/s41551-024-01219-1 38834752

[200] Moore N Chevillet JR Healey LJ McBrine C Doty D Santos J . A microfluidic device to enhance viral transduction efficiency during manufacture of engineered cellular therapies. Sci Rep. (2019) 9:15101. doi: 10.1038/s41598-019-50981-9 31641163 PMC6806008

